# Electron transparent nanotubes reveal crystallization pathways in confinement†

**DOI:** 10.1039/d3sc00869j

**Published:** 2023-05-30

**Authors:** Johanna M. Galloway, Zabeada P. Aslam, Stephen R. Yeandel, Alexander Kulak, Martha A. Ilett, Yi-Yeoun Kim, Angela Bejarano-Villafuerte, Boaz Pokroy, Rik M. Drummond-Brydson, Colin L. Freeman, John H. Harding, Nikil Kapur, Fiona C. Meldrum

**Affiliations:** a School of Chemistry, University of Leeds Leeds LS2 9JT UK f.meldrum@leeds.ac.uk; b Institute for Materials Research, School of Chemical and Process Engineering, University of Leeds Leeds LS2 9JT UK; c Department of Materials Science and Engineering Sir Robert Hadfield Building, Mappin Street Sheffield S1 3JD UK; d Department of Materials Science and Engineering, Technion – Israel Institute of Technology Haifa 3200003 Israel; e School of Mechanical Engineering, University of Leeds Leeds LS2 9JT UK

## Abstract

The cylindrical pores of track-etched membranes offer excellent environments for studying the effects of confinement on crystallization as the pore diameter is readily varied and the anisotropic morphologies can direct crystal orientation. However, the inability to image individual crystals *in situ* within the pores in this system has prevented many of the underlying mechanisms from being characterized. Here, we study the crystallization of calcium sulfate within track-etched membranes and reveal that oriented gypsum forms in 200 nm diameter pores, bassanite in 25–100 nm pores and anhydrite in 10 nm pores. The crystallization pathways are then studied by coating the membranes with an amorphous titania layer prior to mineralization to create electron transparent nanotubes that protect fragile precursor materials. By visualizing the evolutionary pathways of the crystals within the pores we show that the product single crystals derive from multiple nucleation events and that orientation is determined at early reaction times. Finally, the transformation of bassanite to gypsum within the membrane pores is studied using experiment and potential mean force calculations and is shown to proceed by localized dissolution/reprecipitation. This work provides insight into the effects of confinement on crystallization processes, which is relevant to mineral formation in many real-world environments.

## Abstract 2

Transparent nanotubes made by coating track etched membranes with amorphous titania are used to study the evolutionary pathways of calcium sulfate crystallization in confinement.

## Graphical abstract

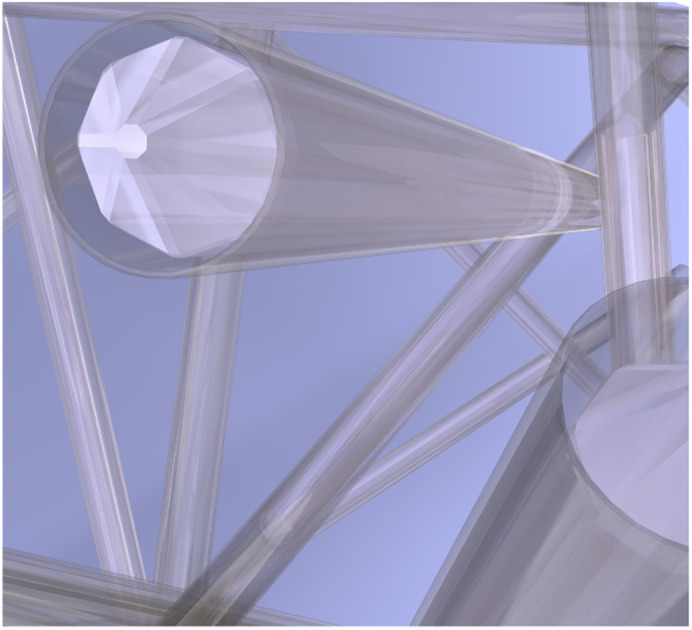

## Introduction

Calcium sulfate attracts considerable attention due to its importance in the environment and industry.^1,2^ In addition to being a key component of the terrestrial sulfur cycle, it is used in enormous quantities as Plaster of Paris,^3^ as a component of fertilizers^4^ and of cement,^5^ and its uncontrolled precipitation contributes to adverse weathering effects^6^ and scale deposition in pipes during water treatment.^7^ Its rich structural chemistry is also key to many applications. Calcium sulfate can exist in three forms according to its degree of hydration: anhydrite (CaSO_4_), bassanite (CaSO_4_·0.5H_2_O), and gypsum (CaSO_4_·2H_2_O).^2^ Each of these phases differs in the characteristic sizes and morphologies of the crystals, and the rapid transition from bassanite to gypsum on addition of water is responsible for the setting of plaster.^3,8^ Whilst a rare mineral on Earth, bassanite is present in significant quantities on Mars, leading to many proposals about its formation mechanisms.^9,10^

Significant efforts have therefore been made to identify the mechanisms by which calcium sulfate forms, and to develop strategies for controlling its crystallization. Gypsum precipitates from aqueous solution under ambient conditions, where it is the thermodynamically stable phase of calcium sulfate at temperatures below 40–60 °C.^1,2,11,12^ Anhydrite becomes the stable phase above this temperature, but gypsum continues to precipitate up to temperatures of ≈95 °C due to the extremely slow crystallization of anhydrite. Bassanite crystallizes at higher temperatures, despite being metastable with respect to both gypsum and anhydrite.^13^ Recent time-resolved studies of calcium sulfate precipitation from aqueous solutions have complicated this picture, where they revealed that bassanite can form as a precursor to gypsum at room temperature under some reaction conditions,^14–21^ but that gypsum forms directly in others.^22^ Bassanite forms in water–alcohol mixtures.^23,24^ It also forms at lower temperatures in high salt concentrations,^2,25^ for example, pure bassanite formed at 60 °C in the presence of 4.3 M NaCl.^13^

In this work we explore the use of confinement to direct the formation of calcium sulfate, and present a novel strategy that enables visualization of the mechanisms by which crystals evolve within nanoscale environments. Confinement has significant effects on crystallization processes, retarding nucleation and growth, stabilizing metastable phases, influencing crystal orientation and determining whether particles are single crystals or polycrystalline.^26,27^ It is also highly relevant to crystallization in the many real-world environments (*e.g.* within porous media) which offer small volumes rather than bulk solution conditions. We have recently shown that precipitation of calcium sulfate within controlled pore glasses (CPGs) that exhibit sponge-like interconnected 7 nm pores leads to the stabilization of bassanite.^28^ However, significant questions remain about how the crystals develop within these settings, and how the crystals are influenced by these confined environments.

Here, the cylindrical pores of track-etched (TE) membranes were used as crystallization environments, where these are available in a wide range of pore diameters, and in contrast to CPGs, have simple shapes. Our results demonstrate that the mineral phase can be selected according to the size of the pores, and that the crystal orientation is defined by the pore geometry. While single crystals of a range of compounds including calcite,^16^ vaterite^29^ aragonite (CaCO_3_),^30^ and hydroxyapatite (Ca_5_(PO_4_)_3_OH)^31^ form in the pores of TE membranes, it has not yet been possible to study individual crystals *in situ* within the pores, meaning that the mechanism by which they develop is unknown. We therefore developed a novel strategy that allows us to study the evolution of crystals within the membrane pores, where mineralization is conducted within membranes coated with a thin layer of amorphous titania. Subsequent membrane dissolution then releases electron-transparent nanotubes that provide mechanical stability for early stage and fragile precursor particles. Finally, we study the transformation of bassanite to gypsum.

## Results

### Calcium sulfate precipitated in bulk solution

**Fig. 1:**
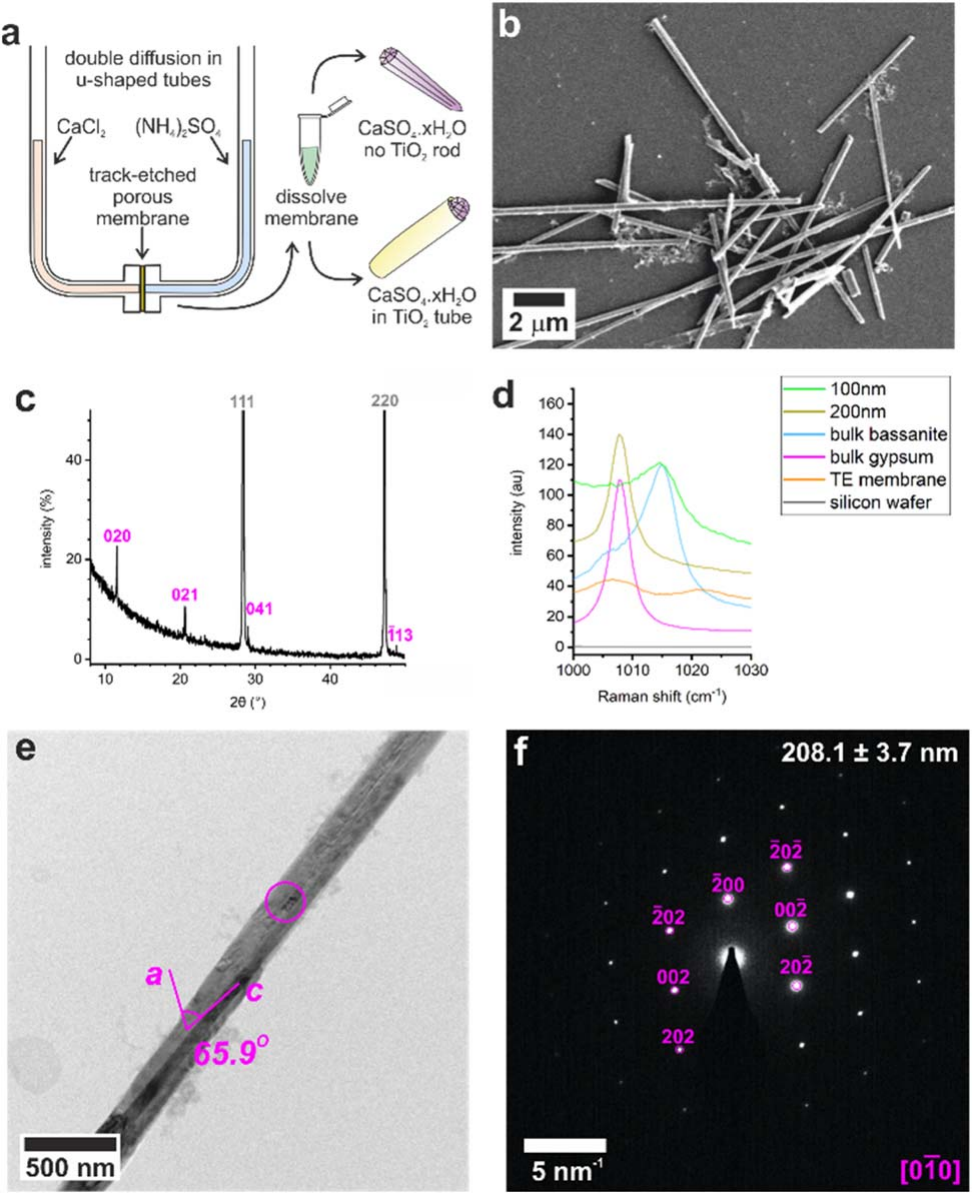
(a) Illustration of U-tube counter-diffusion apparatus used to precipitate calcium sulfate within TE membrane pores. Crystals formed in 200 nm diameter pores analyzed by (b) SEM, (c) p-XRD (silicon powder spike labelled in grey, gypsum in pink), (d) Raman spectroscopy, (e) TEM, and (f) the corresponding SAED pattern. The circle on (e) indicates the area selected for diffraction in (f) which is annotated with the crystal diameter and the fit to gypsum [01̄0].

Control experiments were conducted by precipitating calcium sulfate in bulk solution by combining equal volumes of 3 M aqueous solutions of CaCl_2_·2H_2_O and (NH_4_)_2_SO_4_. This resulted in the rapid formation of gypsum crystals with lath-like morphologies (Fig. S1a†) and average sizes of 3.5 ± 1.6 μm by 0.6 ± 0.2 μm (*n* = 80). Powder X-ray diffraction (p-XRD, Fig. 1b) and Raman spectroscopy (Fig. 1d and S2†) confirmed the gypsum polymorph, where the p-XRD peaks at 2*θ* = 11.66° and 20.77° correspond to the (020) and (021) planes of gypsum, and the Raman peak at 1008 cm^−1^ corresponds to the *ν*_1_ symmetric stretch of sulfate in gypsum.^32,33^ Transmission electron microscopy (TEM) and associated selected area electron diffraction (SAED) showed that the morphological long axis of these crystals was parallel to the [001] axis of gypsum (Fig. S1c and d†).

These crystals were so beam sensitive that it was only possible to obtain indexable SAED patterns at very low electron doses (2.5–5.0 e^−^Å^−2^ per image). Doses of just 30–50 e^−^Å^−2^ per image resulted in distortion and shortening along the gypsum [100] axis, probably due to dehydration (Fig. S3a†), while a short intense burst of electrons was enough to drill holes in the rod and induce transformation to bassanite (Fig. S3b†). More prolonged over-exposure transformed the sample to polycrystalline bassanite and anhydrite (Fig. S3c†). Our analyses indicate that doses of just 30–50 e^−^Å^−2^ per image provided sufficient energy to induce water loss from gypsum under the vacuum of TEM imaging (≈9 × 10^−8^ Torr). Lower doses of 2.5–5.0 e^−^Å^−2^ per image were therefore used to minimize beam damage and sample dehydration during imaging and analysis.

### Calcium sulfate precipitated in track-etched membranes

These results were compared with calcium sulfate precipitated within the confines of the cylindrical pores of 20–25 μm thick polycarbonate TE membranes, with pore diameters of 10, 25, 50, 100, and 200 nm. A schematic of the experimental set-up is shown in Fig. 1a, where counter diffusion of 3 M CaCl_2_·2H_2_O and 3 M (NH_4_)_2_SO_4_ solutions led to precipitation of calcium sulfate within the pores. Mineralization proceeded for 16 h before the polycarbonate membrane was dissolved in dichloromethane and the pore contents to transferred water (Fig. 1b). Previous studies with TE membranes employed an ethanol washing step,^29–31,34,35^ which was omitted here, as ethanol can be used to precipitate bassanite.^23,24^ This led to some residual organics in these samples, which appears as diffuse areas in TEM, but ensures that the mineralogy of our sample is preserved. This methodology is effective in isolating larger, mechanically stable crystals from the membrane.

**Fig. 2:**
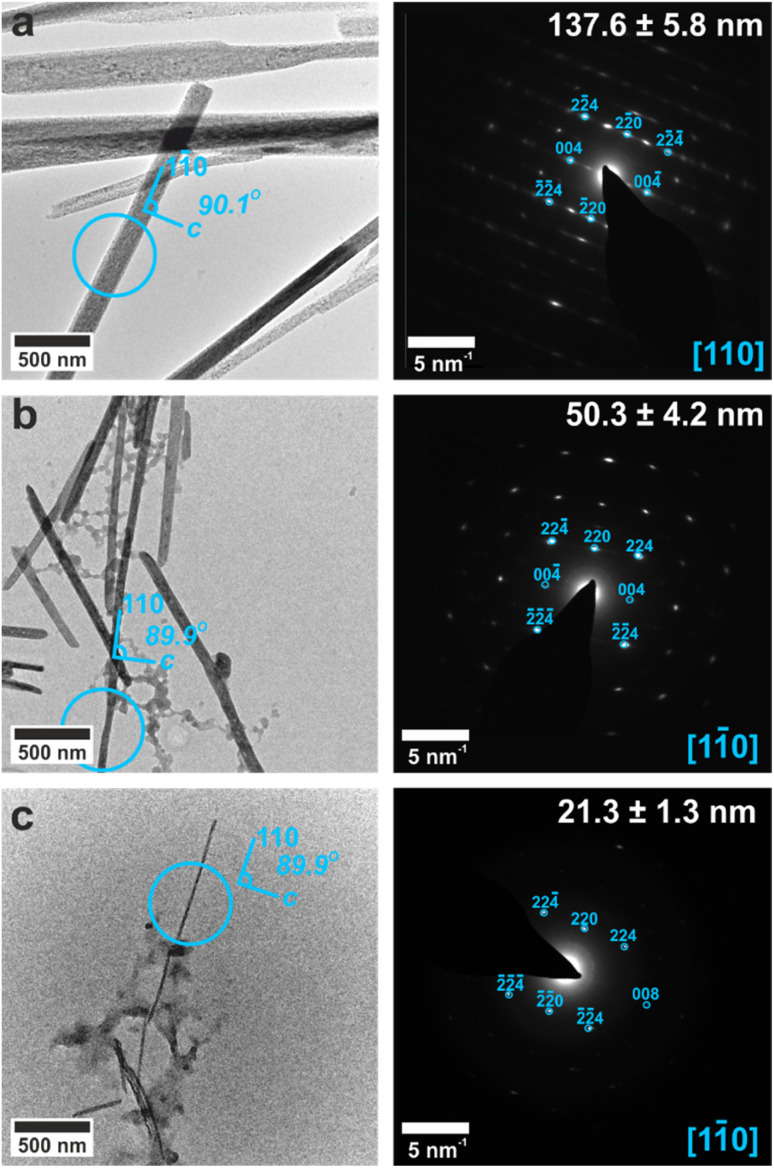
TEM and SAED of calcium sulfate crystallized within (a) 100 nm, (b) 50 nm, and (c) 25 nm pores. The circles on the TEM images (left) show the area selected for diffraction (right) fitted to bassanite 〈110〉.

The intra-membrane crystals exhibited morphologies defined by the cylindrical pores (Fig. 1b). Rods up to ≈20 μm long were isolated from pores with diameters from 25 to 200 nm (Fig. S4†), while nanoparticle aggregates were isolated from the 10 nm pores (Fig. S5a†). The crystals formed in the 200 nm pores were gypsum, as identified using p-XRD (Fig. 2c) and Raman spectroscopy (Fig. 2d and S1†).^32,33^

In contrast, the crystals formed in the 100, 50 and 25 nm pores were single crystals of bassanite (Fig. 2). Sufficient material could be isolated from the 100 nm pores to carry out Raman analysis (Fig. 2d),^32^ while TEM and SAED were used to identify the polymorph formed in the 50 and 25 nm pores. In all cases, the intramembrane bassanite crystals exhibited long axes parallel to the 〈110〉 direction, such that the *c*-axis lies parallel to the short axis (Fig. 2). This contrasts with bassanite crystals precipitated from bulk ethanolic solutions^23^ whose long axes are coincident with the [001] axis (Fig. S6†). These two different crystallographic orientations of bassanite are shown in Fig. S7.†

### Calcium sulfate precipitated in amorphous titania nanotubes

**Fig. 3:**
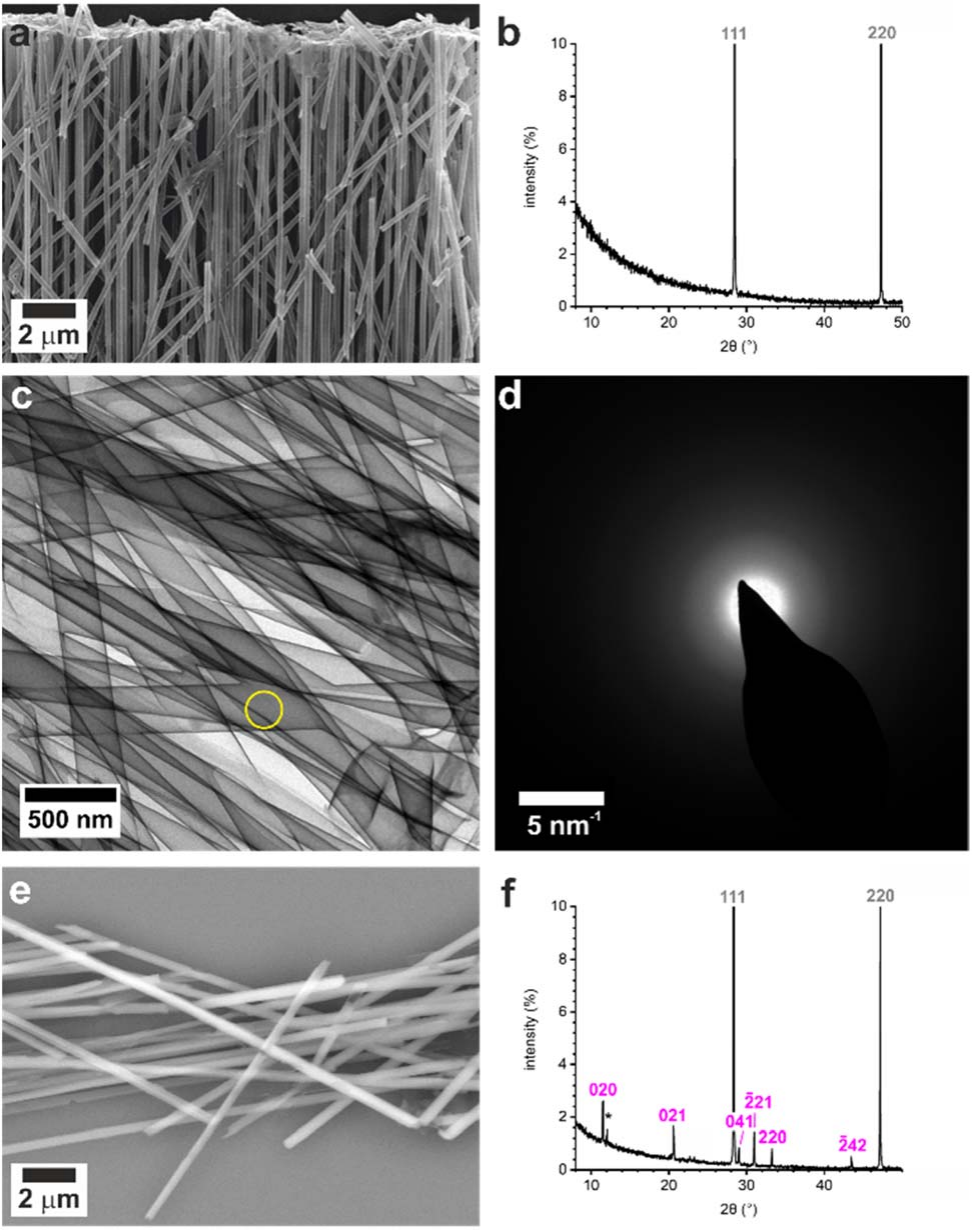
(a) SEM image of unmineralized titania nanotubes and (b) p-XRD pattern shows only reflections from the silicon standard (labelled in grey) confirming their amorphous character. (c) TEM image of unmineralized TiO_2_ tubes from 200 nm pores and (d) corresponding SAED pattern showing that they are amorphous. Area selected for diffraction in (d) indicated by yellow circle on (c). (e) SEM image (CBS detector) of tubes containing calcium sulfate crystals and (f) p-XRD pattern confirming gypsum reflections labelled in pink.

The mechanism by which the crystals form within the membrane pores was investigated by precipitating calcium sulfate within TE membranes that had been coated with a 5–10 nm layer of amorphous TiO_2_ prior to mineralization. Dissolution of the membrane then released titania nanotubes containing the calcium sulfate particles, where the original location and orientation of the crystallites was preserved (Fig. 3e). The titania film was generated using atomic layer deposition (ALD), where the process was optimized to ensure that the film was structurally-competent.^36^ As polycrystalline titania is deposited above 150 °C,^37^ amorphous titania was deposited at 140 °C. The cycle steps are described in Table S1.† Larger diameter pores (50–200 nm) underwent 200 cycles to deposit a TiO_2_ layer, while smaller diameter pores (10–25 nm) were subjected to 100 cycles, as 200 cycles yielded tubes with sealed ends (Fig. S8†).

Dissolution of the membranes released titania nanotubes that maintained their 3D cylindrical form, and SAED confirmed that they were amorphous (Fig. 3, S9 and S10†). The nanotubes were characterized using SEM to measure their external diameters, and TEM images to measure the internal diameters and wall thicknesses (Table S5†). Wall thicknesses approached ≈5 nm after 100 cycles and ≈9 nm after 200 ALD cycles. These values are consistent with an estimated thickness of ≈0.48 Å per cycle from ellipsometry of titania films deposited on a silicon wafer under the same conditions (140 °C, 200 cycles).

**Fig. 4:**
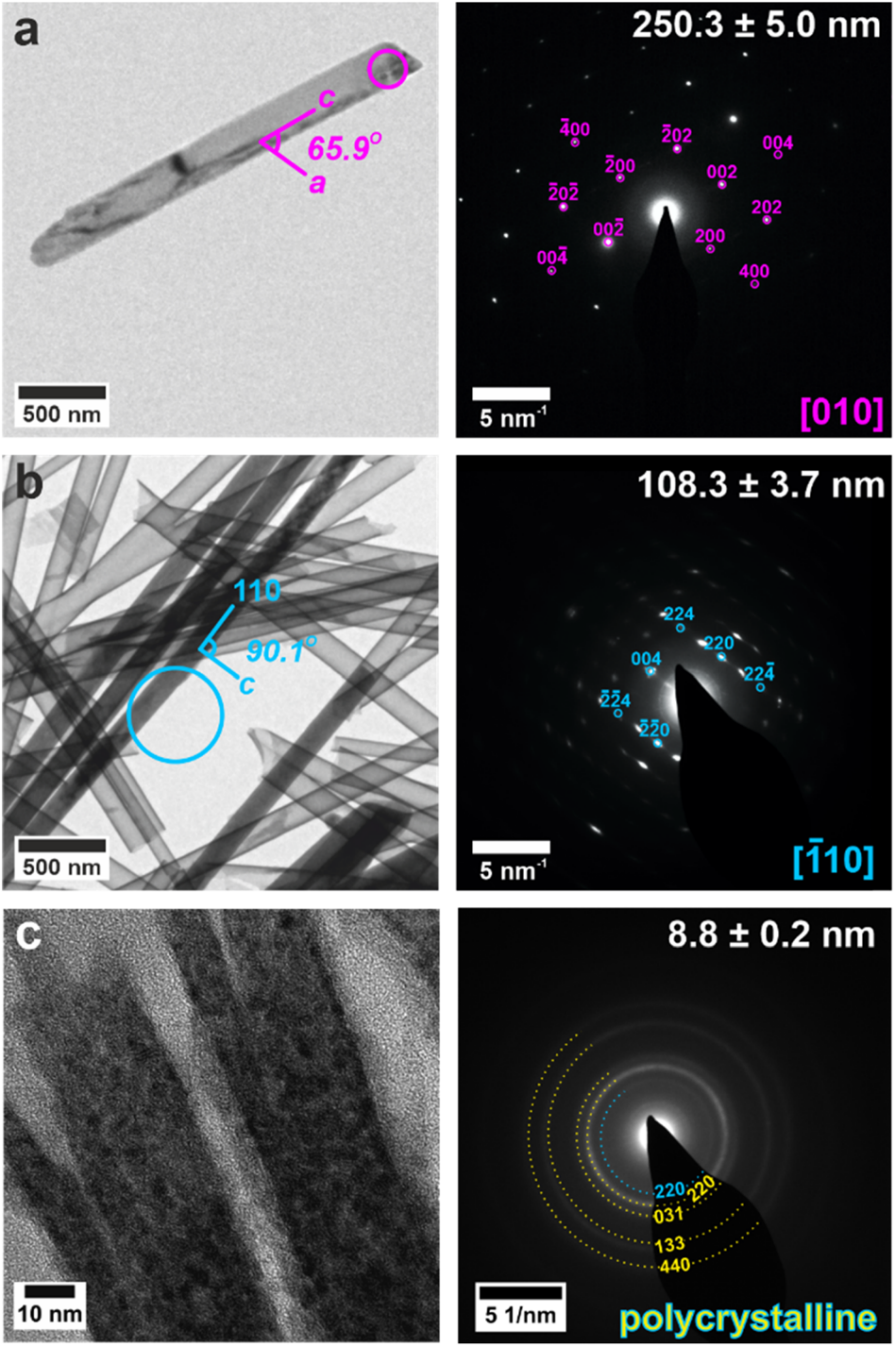
TEM images (left) and SAED patterns (right) of calcium sulfate precipitated within titania nanotubes with pore diameters of (a) 200 nm, (b) 100 nm, (c) 10 nm. TEM images (left) are annotated with a circle indicating the area selected for diffraction. Gypsum reflections are shown in pink, bassanite in cyan and anhydrite in yellow. Measured crystal diameters are annotated on the SAED patterns. An extended version of this Fig. 4 is given in Fig. S13.†

The crystals could be characterized within the titania nanotubes using both SEM and TEM. SEM imaging was performed using a circular backscatter (CBS) detector, as this allowed single crystals to be imaged *in situ* within the nanotubes due to their greater electron density as compared with the TiO_2_ tube walls (Fig. 3e and S11†). Energy dispersive X-ray (EDX) mapping in SEM showed that calcium and sulfur were associated with the crystals within the titania tubes (Fig. S12†), while TEM demonstrated that the mature crystals exhibited the same polymorphs and crystallographic orientations as those released from uncoated TE membrane pores (Fig. 4 and S13†). It was also possible to analyze precipitates formed in the smallest pores (≈10 nm diameter), which indexed to polycrystalline bassanite and anhydrite (Fig. 4c and S13e†). These results are summarized in Table 1.

**Table 1:** Summary of calcium sulfate crystallization in TE membrane pores

Condition	Polymorph	Crystal description	Alignment (long axis)	Alignment 2 (perpendicular axis)
Bulk aqueous	Gypsum	Single crystal laths	001	010
Bulk ethanol + water	Bassanite	Single crystal nanorods	001	100, 010 or {110}
200 nm pore	Gypsum	Single or 2–3 elongated rods	001	010
100–25 nm pore	Bassanite	Single crystal elongated rods	{110}	001
10 nm pore	Bassanite & anhydrite	Polycrystalline particles fill tubes	None	None

### Studying the evolution of calcium sulfate crystals in confinement

**Fig. 5:**
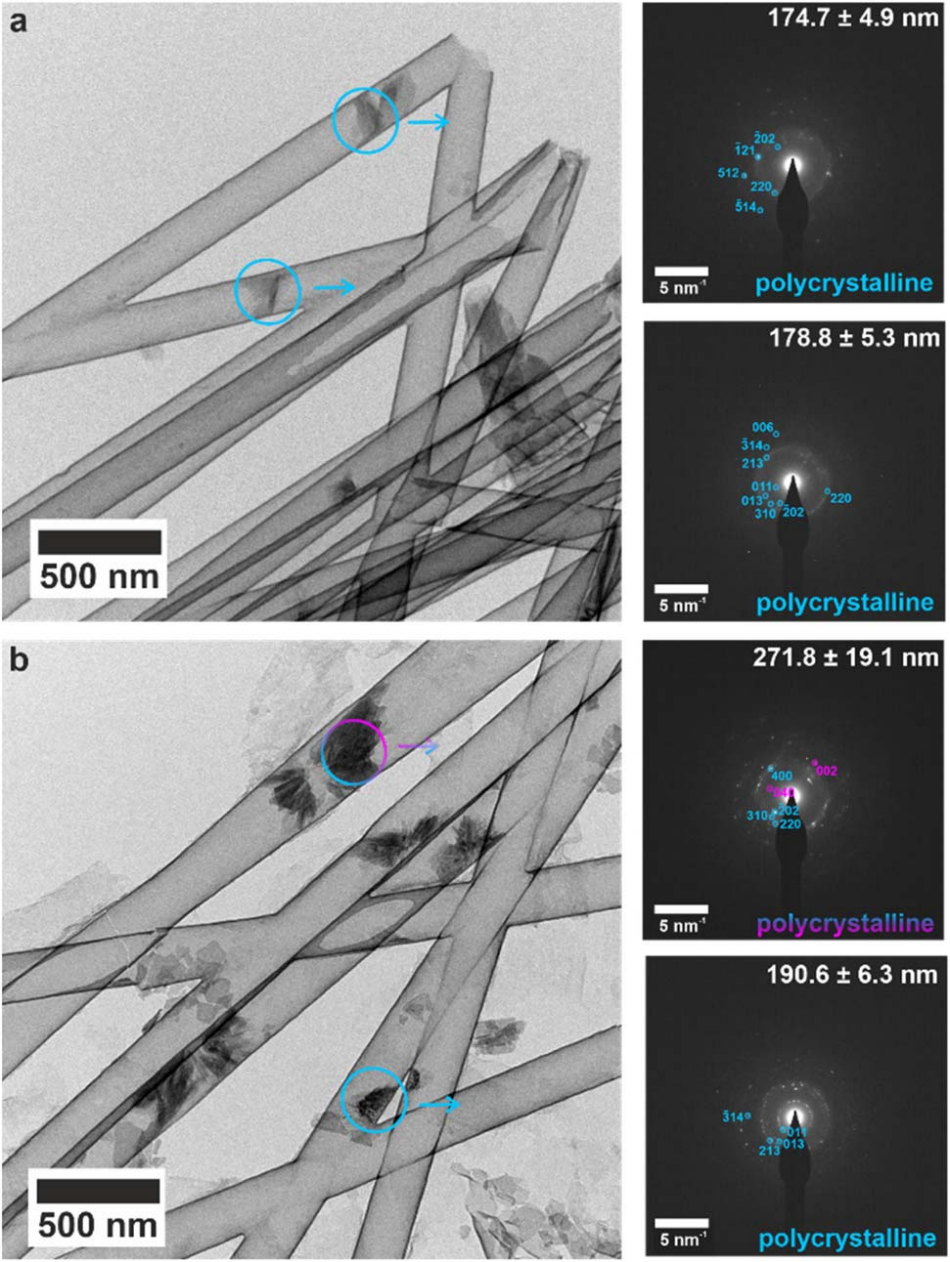
TEM images and corresponding SAED patterns of calcium sulfate precipitated within 200 nm (manufacturer quoted) TiO_2_ nanotubes after 1 h. The area selected for diffraction is indicated by circles on TEM images, and arrows indicate the corresponding SAED pattern. Bassanite reflections are labelled in cyan and gypsum in pink. (a) All small mineral plugs index to polycrystalline bassanite, and (b) slightly larger mineral plugs from same time-point showed both bassanite and gypsum reflections. An extended version of this Fig. 5 is shown in Fig. S18.†

The early mineralization products and their evolution into orientated single crystals was studied by performing crystallization within the titania nanotubes. The nanotubes were isolated after 1, 4 and 16 h of mineralization, and the crystallite sizes were measured using SEM (Fig. S14†), and TEM for 200 nm (Fig. S15†), 100 nm (Fig. S16†) and 50 nm (Fig. S17†) tubes. Most tubes were empty or contained small (<200 nm) electron dense particles after 1 h. Single crystals of bassanite with the same orientation as the mature crystals were observed in the 50 nm tubes (Fig. S17a†), as compared with polycrystalline bassanite particles in the 100 nm and 200 nm nanotubes (Fig. S16a and S17a† respectively). Smaller mineral plugs observed in 200 nm tubes indexed to polycrystalline bassanite (Fig. 5a and S18a†), whereas slightly larger plugs showed a polycrystalline mixture of bassanite and gypsum (Fig. 5b and S18b†). This shows that bassanite formed prior to gypsum in the 200 nm pores.

Analysis after 4 h showed that the average crystal lengths had increased to 300–700 nm, and were greater in larger pores (Fig. S15b, S16b and S17b†). Importantly, multiple mineral plugs were observed in many nanotubes, which demonstrates that the single crystals generated after longer incubation periods evolve from many crystallites rather than a single nucleation event. Single crystals of bassanite were present in the 100 nm and 50 nm pores and were oriented with the 〈110〉 axis parallel to the long axis of the pore, while small gypsum rods were present in the 200 nm pores and were oriented with the [001] axis aligned with the long axis of the nanopore (Fig. S15b, S16b and S17b†). The orientations of the mature bassanite and gypsum crystals were therefore established at early time points.

After 16 h, the length of the mineral rods were 3.7 ± 2.6 μm in 50 nm nanotubes, 6.4 ± 5.2 μm in 100 nm nanotubes, and 9.2 ± 5.8 μm in 200 nm nanotubes. That the gypsum and bassanite rods were true single crystals rather than oligo-crystalline was demonstrated by recording SAED patterns along the entire rod lengths (Fig. S15c, S16c and S17c†). Given that multiple crystals were observed at early times, this suggests that an Ostwald ripening process may occur within the pores to give single crystal products as the crystallization fills the confines of the pores.

### Maintaining confinement stabilizes bassanite

**Fig. 6:**
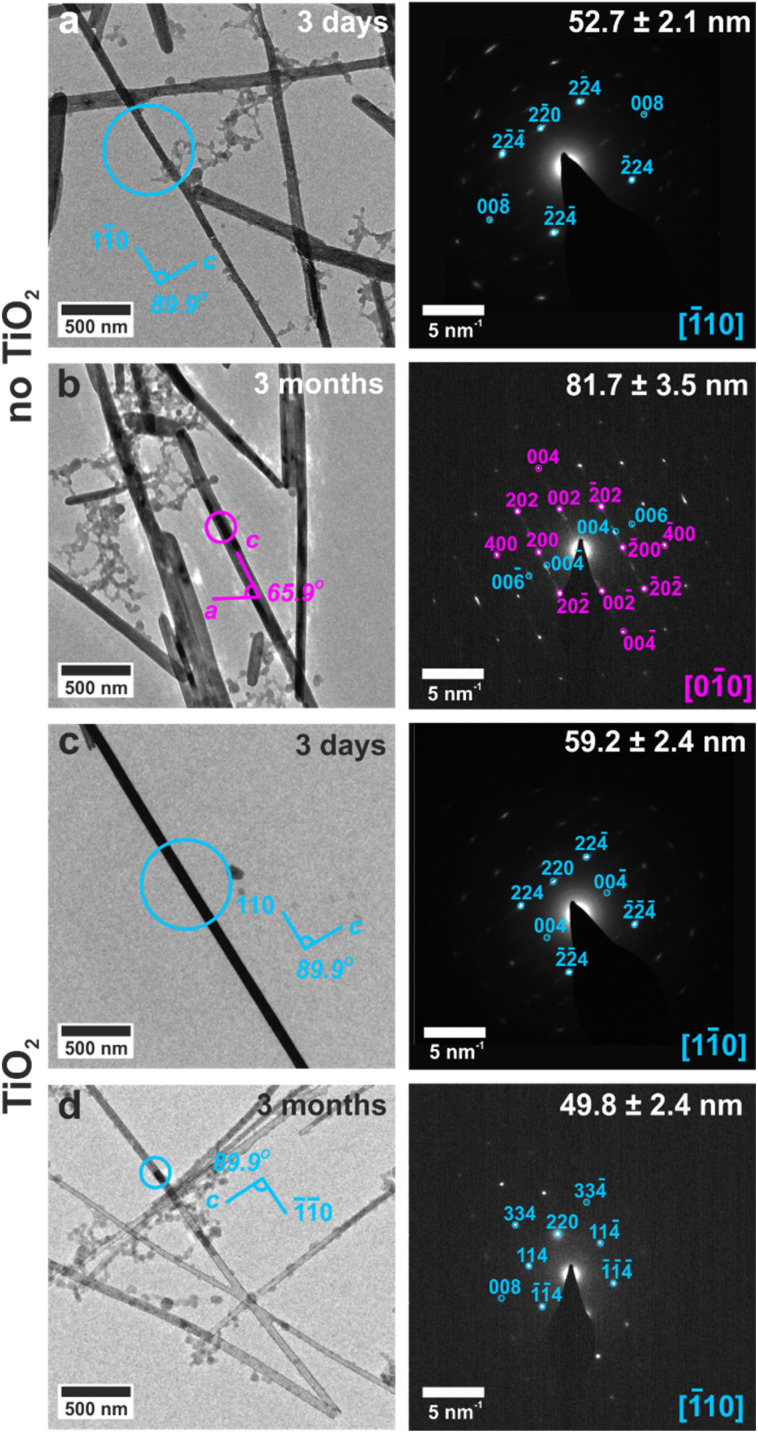
TEM images and corresponding SAED patterns of calcium sulfate precipitated within 50 nm (manufacturer quoted) TE membrane pores before and after aging for 3 months in air. The area selected for diffraction is circled on the TEM image, gypsum reflections are labelled in pink and bassanite in cyan. In the absence of a titania nanotube, the templated crystals are (a) bassanite, but (b) transform to gypsum within 3 months. In the presence of a titania coating (c) the original bassanite crystals (d) remain unchanged after 3 months.

The ability of these confined systems to stabilize metastable bassanite was also studied. Membranes containing bassanite crystals were left in contact with the mineralization solution for 1 month, after which time all intra-membrane crystals had dissolved. This may be due to Ostwald ripening^38^ of larger, unconfined crystals that deposit in the U-tube arms and then grow at the expense of the smaller intra-membrane crystals. 25–100 nm diameter nanotubes containing bassanite crystals were also isolated, transferred to TEM grids, and were imaged before and after storing the grids under ambient conditions for 3 months. No change in the crystals was observed, where they remained as single crystals of bassanite (Fig. 6d, S19d and S20d†). This can be attributed to the vast majority of the surface being protected from the environment by the titania coating.

In contrast, bassanite crystals that were isolated from uncoated TE membranes transformed to gypsum when stored on TEM grids under ambient conditions for 3 months. The original size and shape of the bassanite crystal was maintained, and the long axis corresponded to the [001] axis of gypsum (Fig. 6b, S19b and S20b†). As a solid state transformation of bassanite to gypsum would keep the [001] axes coincident, the transformation is attributed to a local dissolution/reprecipitation process, consistent with recent liquid cell and cryo-TEM work.^22^ These results are summarized in Table S4.† As a final comparison, bassanite nanorods precipitated from ethanolic solution remained as bassanite when stored on TEM grids for 18 months (Fig. S6c and d†). This may be due to their crystallographic orientation (*c*-axis aligned with the long axis of the control rods rather than perpendicular alignment of the confined rods) or the ethanolic synthesis leading to a more stable bassanite surface structuring.^39,40^ Either route could inhibit water from entering into the bassanite structure of the control particles, thus stabilizing them against transformation to gypsum.

### Modelling water in CaSO_4_·*x*H_2_O structures

**Fig. 7:**
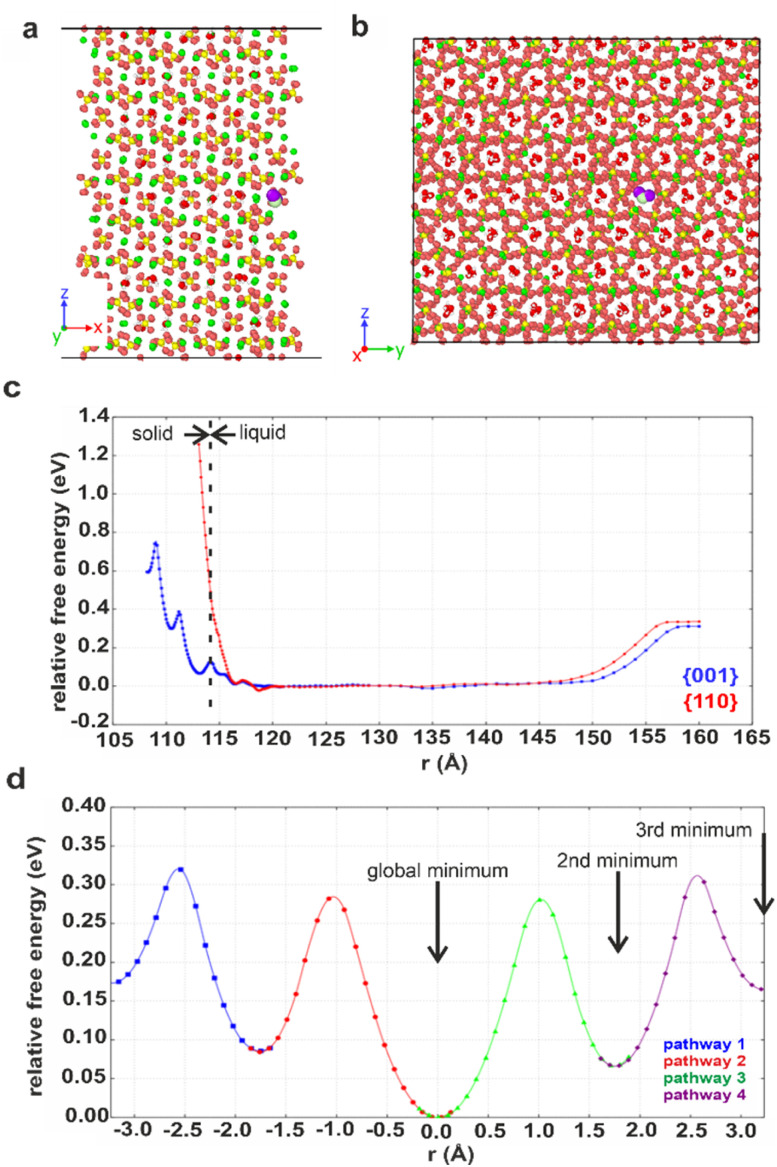
Snapshots showing the (001) surface of bassanite viewed from (a) the side and (b) the top. Structural water (white and red) in channel pores between the calcium sulfate units (Ca green, S yellow and O orange). A water molecule from solution (white and purple, others have been removed for clarity) nestled in a structural water channel in a 113 Å minimum. (c) Graph of free energy barriers to bringing water into the bassanite structure through the {001} (blue) and {110} (red) surfaces. The water molecule from solution is either next to the surface (left) and enters the “bulk” water surrounding the crystal (central plateau) or is extracted from the water layer to vacuum (right). (d) Free energy barriers to migrations of an interstitial water. Each color represents a different interstitial migration path and the distance the interstitial has travelled. Each pathway is truncated where the next water molecule is displaced to show one fully periodic pathway through the cell. Positions of the water molecules within the pores are shown in Fig. S22.†

Computational methods were applied to study possible hydration mechanisms of bassanite,^41^ where potential of mean force (PMF) calculations were performed using molecular dynamics (MD) simulations. The PMF was used to extract the free energy for a water molecule in solution adjacent to the {001} surface of bassanite on which the open ends of the water channels terminate, and to the {110} surface, which does not have open water channels (Fig. S7†). Calculation of the free energy barriers to bringing water into the bassanite structure confirmed that there are multiple minima as a water molecule approaches the {001} surface, with minima at 0.1 and 0.4 eV (Fig. 7c). A much higher energy barrier of at least 1.2 eV was observed at the {110} surface. These calculations show that water is able to freely enter the bassanite structure from solution at the faces presenting water channels. For bassanite formed from a bulk ethanolic solution, the {001} surface is small as it is expressed on the short ends of the rods, so very few open water channels are accessible. However, for bassanite formed in confinement, the {001} faces lie along the side of the rod (Fig. S7†). Upon release from confinement, these bassanites display many more open water channels than those formed from a bulk solution, facilitating the ingress of water into the bassanite formed in confinement.

The mechanism by which water migrates within bassanite was then investigated. Considering first interstitial hopping, analysis showed that in addition to the three sites occupied by water in the bassanite structure, there are three unoccupied sites within the water channels. An interstitial water molecule occupying a vacant site can displace a crystallographic water molecule, creating a new interstitial water further along the channel. This enables water to migrate throughout the bassanite crystal (Fig. 7d), with a barrier to migration of ≈0.3 eV. Water migration was also considered as a Frenkel pair,^42^ where an interstitial water and a vacancy are formed, and the vacancy migrates to the crystal surface allowing solution water to enter the structure (Fig. S21 and S22†). However, this has a higher energy barrier of ≈0.5 eV, indicating that interstitial hopping is the most energetically favorable method for water to enter the bassanite structure.

## Discussion

Our results are summarized in Table 1 and demonstrate that bassanite is the first phase formed in membrane pores of diameters 25–200 nm, and subsequent transformation to gypsum occurs within 4 hours in the 200 nm pores. A mixture of polycrystalline bassanite and anhydrite formed in the smallest 10 nm pores. The initial formation of a metastable phase in the membrane pores is consistent with the behavior of a wide range of inorganic^29–31,34,35,43^ and organic^44–46^ systems in confinement. This has been attributed to effects including the exclusion of polymorphs with critical nuclei larger than the confining pore size^47–49^ and changes in the relative stabilities of different polymorphs as small particle sizes.^46^ The exclusion of impurities,^26,27,50,51^ minimal topographic defects that can act as favorable nucleation sites in bulk systems,^52,53^ and the reduction in nucleation rates in small volumes can also influence the supersaturation at which nucleation occurs in some systems.

**Fig. 8:**
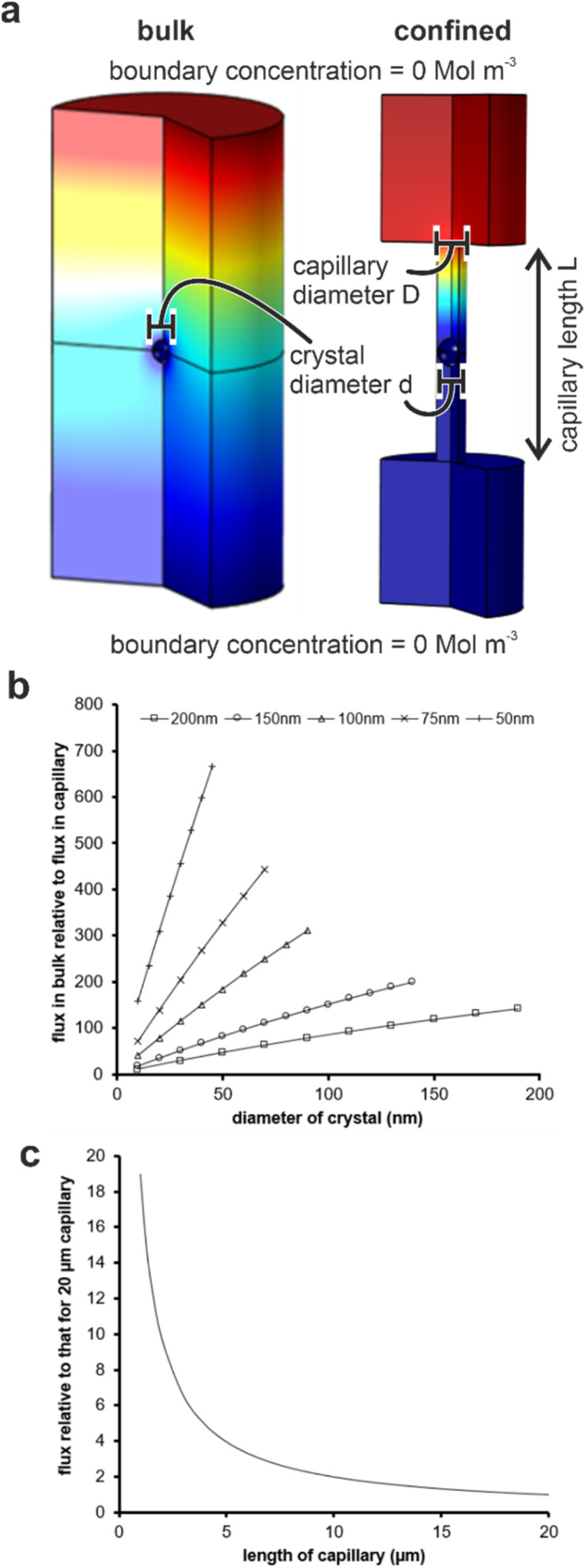
(a) Simulation conditions for evaluating flux transport in (left) bulk solution and (right) a capillary. (b) Plots of flux transport to a crystal in bulk solution normalized to one of identical size located at the center of a 20 μm long capillary with five different capillary diameters (key on graph). (c) Mean flux transport to a crystal located in the center of a capillary of a specified length relative to the same crystal located in the middle of a 20 μm capillary. Scaled simulations for capillaries of diameter 50–200 nm all collapse onto 1 curve (within 10%).

Material transport is significantly reduced in the membrane pores as compared with bulk solution due to geometric hindrance and the elimination of advection. Finite element simulations were performed using COMSOL to estimate the impact of confinement on the flux to a growing crystal. An individual crystal with a defined diameter was placed at the center of a cylindrical capillary, whose ends were connected to large reservoirs (Fig. S8a†) and the flux of ions to the crystal was then compared to that of an unconfined crystal of the same dimensions immersed in a bulk solution. An increase in the scaled flux (the flux to the unconfined crystal relative to the flux to the crystal in the capillary) corresponds to a decrease in the flux to the crystal in the capillary. Fig. 8c shows the influence of the capillary length on the flux transport to the crystal surface.

For each capillary, increasing the diameter, and thus the amount of solution exposed to a given surface area of the crystal, results in a greater transport of ions. Conversely, a reduction in the diameter of the capillary for any given crystal diameter reduces the flux due to geometric hindrance. The length of the capillary also influences the flux, where calculations were made by comparing the flux to a crystal placed in the middle of a capillary of selected diameter to one situated in a capillary of length 20 μm (Fig. 8c). Longer capillaries are seen to hinder the transport of ions due to a reduction in the diffusion gradient between the reservoir and the crystal. Simulations were performed for capillaries with diameters of 50, 100 and 200 nm containing crystals that were 95 and 50% of the capillary diameter, and all scaled simulations collapse within ±10%. The most significant observation from these simulations is the magnitude of the geometric hindrance. The smaller diameter (50 nm) and longer (20 μm) capillaries have a reduced flux of almost 1000 times that which would be observed in the bulk. There was a still significant reduction of 150 times for the 200 nm diameter capillaries.

Reduced flux is typically associated with a slower build-up of supersaturation and lower threshold supersaturations, which generally favors the formation of stable polymorphs, as observed for crystallization in confinement in gels.^54^ Therefore, the initial nucleation of bassanite rather than of gypsum within the pores does not arise from reduced flux. Reduced flux may also reduce the rate of transformation of metastable polymorphs to more stable ones. Metastable phases are often very short lived in bulk solution,^28^ and a sequence of increasingly more stable polymorphs are seen prior to the formation of the stable end-product.^16,55,56^ This is consistent with our experimental observations, where bassanite is the first phase formed in all of the pore sizes studied. So it may be that the reduced flux reduces the rate of transformation of gypsum to bassanite, thereby stabilizing bassanite in the smaller pores.

While it is tempting to attribute the observation of bassanite here to the capture of an intermediate polymorph, it is noted that both bassanite and gypsum have been observed within the confines of the 7 nm pores in CPG rods.^28^ Bassanite precipitated within unfunctionalized CPG rods, and was stable for over 3 weeks, whereas both bassanite and gypsum formed within carboxylate functionalized rods. Further, in CPGs the gypsum particles seemed to form directly rather than transforming from bassanite. Therefore, the initial formation of bassanite in the membrane pores appears to be dominated by the interaction of the nascent nuclei with the pore wall, an effect that will become of increasing importance as the pore diameter decreases, and the surface area to volume ratio increases.

This is consistent with our current understanding of the nucleation mechanisms of calcium sulfate. *In situ* small and wide angle X-ray scattering (SAXS/WAXS) studies of bulk solutions suggested that initially, primary clusters 1–3 nm in size form through the co-assembly of Ca^2+^ and SO_4_^2−^ ions.^20,39,57^ Similar sized clusters have also been observed in MD simulation of supersaturated solutions of calcium sulfate.^58^ These domains collapse to form amorphous aggregates that subsequently reorganize to form gypsum, bassanite or anhydrite according to the reaction conditions.^1,20,39^ Transformation of bassanite to gypsum in solution subsequently typically occurs *via* dissolution/reprecipitation.^22^ The observation of nanoscale co-aligned domains in natural calcium sulfate crystals^1,40^ support that the multiple (bassanite) nucleation events followed by maturation into a larger aligned crystal observed in our confined pores are unlikely to be an artefact of confinement, but instead are something that occurs across scales in the calcium sulfate system.

Considering then the orientation of the crystals formed within the membrane pores, small bassanite crystallites were observed in all pore sizes, and became orientated at early stages of mineralization. Those in the 200 nm pores subsequently transformed to orientated gypsum, likely by a localized dissolution/reprecipitation mechanism. A similar mechanism was observed recently in a time-resolved TEM study of calcium sulfate crystallization in bulk solution, where the initial formation of bassanite rods was followed by gypsum nucleating on the ends of the rods.^21^ The orientation of crystals in anisotropic environments can occur due to competitive growth effects.^30,31,47^ If a crystal is strongly anisotropic, nuclei that are orientated with their fast growing axis parallel to the long axis of the pore will grow unimpeded at the expense of crystals in other orientations *via* Ostwald ripening.^38^ However, as orientated gypsum and bassanite are observed at early reaction times when few crystals are present, this suggests that competitive growth is unlikely to be active in the membrane pores. The pore surface is therefore likely to be responsible for orienting the crystals in confinement.

Both the uncoated and TiO_2_ coated TE membranes were rendered hydrophilic prior to use by plasma treatment,^59^ and at the near neutral pH of calcium sulfate solution (estimated as pH 7.12 using Visual MinTEQ), these surfaces should be relatively uncharged. This may facilitate the nucleation of apolar, water rich faces of calcium sulfate crystals over polar faces,^60–62^ leading to *c*-axis alignment of the confined bassanite perpendicular to the long axis of the pore. Recent calculations^61^ showed that entropy plays a significant role in determining the interfacial free energy of surfaces in the calcium-sulfate-hydrate system, with the entropy contribution to stabilizing the bassanite surfaces being greater than for the gypsum surfaces. This suggests that bassanite is more easily stabilized by attachment to surfaces, such as the pores walls, than gypsum. The bassanite crystals formed in the pores have a different orientation from those formed from ethanol, where the [001] axis is parallel to the long axis of the bulk precipitated nanorods. In the case of gypsum, the faces are all apolar, and the water channels run perpendicular to the [001] axis.^60,61^ The hydrophilic apolar pore wall will therefore facilitate the orientation of gypsum with its *c*-axis parallel to the long axis of the pore, as observed in this work.

It is also interesting to consider the crystallographic relationship between the intra-membrane bassanite crystals and the gypsum crystals into which they transform, where the [001] axes of bassanite and gypsum lie perpendicular and parallel to the long axis of the pores respectively. This was observed during the evolution of bassanite to gypsum in the 200 nm pores, and during the transformation of bassanite crystals isolated from uncoated TE membranes to gypsum in air. The solid-state transformation between bassanite and gypsum is reported to occur with the retention of the *c*-axis.^14,63^ Our PMF calculations show that water is most likely to enter the bassanite through open ends of the water channels on the {001} surfaces by overcoming a 0.4 eV barrier, and then migrate through these channels by interstitial water migration (0.3 eV barrier).

However, direct hydration may be outcompeted by local dissolution/reprecipitation, as was observed experimentally^21,22^ and in simulations.^64^ The greater abundance of accessible water channels in bassanite formed in the confined pores may facilitate faster hydration and subsequent dissolution/reprecipitation when it is released from confinement than was seen for bassanite formed from a bulk solution.

## Conclusions

It is well recognized that confinement can have significant effects on crystallization processes, resulting in the stabilization of metastable phases, the orientation of crystals with respect to the dimensions of the confining volume, and control over morphologies.^26,27^ Notably, these can be observed over length-scales ranging from a few nanometers (the size range of critical nuclei) to hundreds of nanometers. However, unravelling the origins of many of these effects has proven challenging, as it is typically very difficult to visualize the nucleation and growth of crystals within confined volumes. Indeed, while it has previously been shown that the oriented single crystals of a range of compounds are formed within linear pores,^29–31,34,35,43–47,62^ the inability to characterize individual crystals *in situ* within the pores made it impossible to determine the mechanisms by which they form. Here we show that electron transparent nanotubes can be generated by coating the membranes with amorphous TiO_2_, where these protect small intramembrane crystals, and enable us to study the evolution of the crystals. Our results answer long-standing questions about crystallization in these confined volumes by showing that the high aspect ratio single crystals of gypsum and bassanite develop from multiple nuclei, and that orientation is defined by favorable interactions with the pore walls. Systematic studies of confinement effects, such as those described here, will enable the development of strategies that use confinement to control crystallization as well as providing insight into crystallization in many natural environments.

## Methods

### Sample preparation

5–10 nm amorphous TiO_2_ was deposited onto TE membranes (it4ip, BE) using a Cambridge Nanotech Fiji F200 atomic layer deposition (ALD) system, conditions shown in Table S1.† A 1 cm^2^ piece of TE membrane (coated or uncoated) was plasma treated, soaked in ethanol (EtOH, 2 min), then water (2 min) before sandwiching and sealing into the U-tube apparatus (Fig. 1a). 1 mL of each half of the mineralization solution was added to each arm – 3 M CaCl_2_ and 3 M (NH_4_)_2_SO_4_ – and incubated for up to 16 h. Crystal rods or nanotubes were released by dissolution of the membrane in dichloromethane (DCM), washed and transferred into water. Control bulk gypsum was precipitated by mixing the two mineralization solutions (3 M CaCl_2_ and 3 M (NH_4_)_2_SO_4_), and control bulk bassanite was precipitated from an ethanolic solution as per Tritchler *et al.* (2015).^23^

### Sample characterization

Samples were dried onto clean silicon wafers and were imaged using scanning electron microscopy (SEM) in an FEI Nova 450 NanoSEM using a circular backscatter detector (CBD) at 5 keV, and energy dispersive X-ray (EDX) maps were recorded using a Bruker SDD-EDS detector at 18 keV. For transmission electron microscopy (TEM), samples were dried onto TEM grids (formvar-carbon coated copper 200 mesh, EMS, USA). TEM images and selected area electron diffraction (SAED) patterns were collected using an FEI Tecnai TF20: FEGTEM equipped with a Gatan Orius SC600A CCD camera operating at 200 keV using a spot size of 6 (30–50 e^−^ Å^−2^ per image). Low dose images and SAED patterns were recorded using an FEI Titan3 Themis 300: S/TEM with S-TWIN objective lens at 300 keV and set to a screen current of 0.1–0.2 nA (2.5–5.0 e^−^ Å^−2^ per image).

For p-XRD and Raman, samples were dried onto clean silicon wafers and diffraction data were collected using a Brucker-AXS D8 series diffractometer (Cu Kα source), and processed using Bruker-AXS Commander and EVA software. Raman spectra were collected using a Horiba LabRAM HR Evolution microscope using a green 532 nm 50 W laser using LabSpec 6 software. Images were processed using Gatan Microscopy Suite Digital Micrograph version 3.30.2016.0 and/or Fiji^65,66^ version 1.151n_x64-x86. Diffraction standards were obtained from the American Mineralogist Crystal Structure Database (AMCSD):^67^ #4651 gypsum, #6909 bassanite, #5117 anhydrite, and #15108 silicon (Table S2†). SAED were fitted with simulations from these references using SingleCrystal™ ver 2.3.3.

### Computational studies

The potential model of Byrne *et al.* (2017)^68^ was used to produce the free energies of water transport in bassanite. The structure of bulk bassanite was built based on AMCSD #6909.^69^ {001} and {110} surfaces were generated using the METADISE code.^70^ MD simulations were performed using the LAMMPS code^71^ using the dipole correction of Ballenegger *et al.* (2009).^72^ Lattice equilibration was performed in an NPT ensemble using a Nosé–Hoover thermostat and barostat^73,74^ (300 K and 0 bar). Water molecules were inserted or removed as required.

For potential mean force (PMF) calculations, an atom was restrained in a harmonic well and the force applied by the well was recorded during an MD simulation in order to calculate the free energy profile associated with the pathway.^41^ These simulations were performed in an NVT ensemble using a Langevin thermostat^75^ and integration of the average force to obtain the free energy profiles was performed using the trapezoidal rule.

To analyze the flux to a growing crystal, the advection–diffusion equation was solved using COMSOL Multiphysics (ver. 5.5). This was done for (1) confinement within pores by using two large reservoirs connected by a narrow cylindrical channel with a crystal positioned at its center and (2) for a bulk solution using a crystal in the middle of a large reservoir. A concentration of 1 mol m^−3^ and 0 mol m^−3^ were assigned to the top and bottom of the large reservoirs (Fig. S8a†). Initially, the concentration at the surface of the crystal was also set to 0 mol m^−3^. A diffusion coefficient of 1 × 10^−9^ m^2^ s^−1^ was used throughout and was taken as representative of the diffusion coefficients of 0.79 × 10^−9^ m^2^ s^−1^ for Ca^2+^ and 1.10 × 10^−9^ m^2^ s^−1^ for SO_4_^2−^,^76^ and the properties of the fluid taken as water.

## Data availability

The data associated with this publication are openly available from the University of Leeds Data Repository https://doi.org/10.5518/1265.

## Author contributions

The manuscript was written through contributions of all authors. All authors have given approval to the final version of the manuscript. JMG, BP & FCM designed the study. JMG synthesized the samples, collected and analyzed Raman and XRD data and drafted the manuscript; JMG & AB-V synthesized and characterized TiO_2_ coated TE membranes; JMG, ZPA, MI, Y-YK & RMD-B collected and analyzed TEM & SAED; JMG & AK collected and analyzed SEM & EDX; SRY, CLF & JHH set up and analyzed PMF and MD simulations, NK set up and analyzed COMSOL flux models.

## Conflicts of interest

There are no conflicts to declare.

## Supplementary Material

SC-014-D3SC00869J-s001
