# Supplementary material for: Electron transparent nanotubes reveal crystallization pathways in confinement
Source: Chem Sci. 2023 May 30;14(24):6705–15. doi: 10.1039/d3sc00869j (PMC10283488; doi:10.1039/d3sc00869j)
Supplement: SC-014-D3SC00869J-s001 [file SC-014-D3SC00869J-s001.pdf]

Electronic Supplementary Material (ESI) for Chemical Science.
This journal is © The Royal Society of Chemistry 2023

SI: Electron Transparent Nanotubes Reveal Crystallization Pathways in Confinement

J.M. Galloway, Z.P. Aslam, S.R. Yeandel, A. Kulak, M.A. Ilett, Y.-Y. Kim, A. Bejarano-Villafuerte, C.L. Freeman, J.H. Harding, R.M. Drummond-Brydson, N. Kapur, B. Pokroy, F.C. Meldrum

Contents

Extended Methods 2
- **Materials** 2
- **TiO_2 Deposition on TE Membranes** 2
- **Calcium Sulfate Precipitation within TE membranes** 2
- **Bulk Ethanolic Precipitation of Bassanite** 2
- **Sample Characterization** 3
- **Computational Studies** 3

Figure S1. Gypsum crystals from a bulk solution. 5
Figure S2. Raman spectra of calcium sulfate precipitates. 6
Figure S3. TEM and SAED of beam damaged and control gypsum samples. 7
Figure S4. SEM images of calcium sulfate rods formed in TE pores. 8
Figure S5. SEM and TEM of calcium sulfate crystallized within 10 nm pores. 9
Figure S6. TEM and SAED of bulk ethanolic bassanite. 9
Figure S7. Diagram of major crystallographic orientations for bassanite. 10
Figure S8. TEM of TiO_2 deposited using 200 cycles of ALD on 25 nm TE pore 11
Figure S9. SEM of TiO_2 tubes after membrane removal. 12
Figure S10. TEM and SAED of TiO_2 tubes. 13
Figure S11. SEM of calcium sulfate rods mineralized in TiO_2 coated pores. 14
Figure S12. SEM and EDX of TiO_2 tubes mineralized with calcium sulfate. 16
Figure S13. TEM and SAED of calcium sulfate precipitated within titania tubes 18
Figure S14. SEM images of calcium sulfate formed within TiO_2 pores 18
Figure S15. TEM and SAED TiO_2 200 nm pores after 1-16 hours mineralization. 19
Figure S16. TEM and SAED TiO_2 100 nm pores after 1-16 hours mineralization.. 20
Figure S17. TEM and SAED TiO_2 50 nm pores after 1-16 hours mineralization. 21
Figure S18. TEM and SAED TiO_2 200 nm pores after 1 hour mineralization 22
Figure S19. TEM and SAED before and after aging for 3 months – 100nm pores 23
Figure S20. TEM and SAED before and after aging for 3 months – 25nm pores 24
Figure S21. Graphs of energy barriers for migration through bassanite water channels. 25
Figure S22. Snapshots of water in bassanite structures. 26
Table S1. Optimized recipe for TiO_2 deposition within TE membrane pores. 27
Table S2. Unit cell parameters from AMSCD database entries for calcium sulfate polymorphs 28
Table S3. Measurements of unmineralized TiO_2 tubes after membrane removal. 28
Table S4. Summary of calcium sulfate crystallization in TE membrane pores when aged. 29
Table S5. Calcium sulfate rod diameters. 30
References 31

Extended Methods

Materials

All aqueous solutions were made using ultrapure water (18.2 MΩ.cm at 25 °C). Uncoated polycarbonate track-etched membranes were purchased from it4ip (Louvain-la-Neuve, BE). Calcium chloride dihydrate (≥99 % $CaSO_4 \cdot 2H_2O$), ammonium sulfate (≥30 % NH_3 $(NH_4)_2SO_4$) and silicon powder (-325 mesh, 99% trace metals basis) were purchased from Sigma Aldrich (Gillingham, UK), PTFE tape, ethanol (EtOH) and dichloromethane (DCM) from VWR (Lutterworth, UK), and 95+ % sulfuric acid and 30 % hydrogen peroxide from Fisher Scientific (Loughborough, UK). TEM grids were purchased from Electron Microscopy Sciences (EMS, Hatfield, PA, USA), and SEM stubs and copper tape from Agar Scientific (Stanstead, UK).

TiO_2 Deposition on TE Membranes

Titania coatings were deposited in a Cambridge Nanotech Fiji F200 Atomic Layer Deposition (ALD) System in a class 100 cleanroom. The tetrakis(dimethylamido)titanium (TDMAT) precursor was preheated to 75 °C. TE membranes were heated to 140 °C within the deposition chamber at 1 x 10^{-5} torr. The deposition recipe is shown in Table S1. 100 cycles were used to deposit ≈5 nm TiO_2 on the 10 nm and 25 nm diameter pores, while 200 cycles deposited ≈10 nm TiO_2 in the 50-200 nm diameter pores. These thickness of TiO_2 deposited was checked by depositing under the same growth conditions on to silicon wafer (200 cycles = 9.6 nm) using a Woollam M-2000XI ellipsometer.

Calcium Sulfate Precipitation within TE membranes

Glassware was piranha cleaned by immersing in a solution of 7 parts 95+ % sulfuric acid and 3 parts 30 % hydrogen peroxide for >2 hours, rinsed 5 times with water, then dried with a stream of air. A 1 cm^2 membrane was cut out and plasma cleaned in air for 2 minutes (Atto plasma cleaner, Diener). The clean membrane was then immersed in EtOH (2 minutes), then water (2 minutes) before mounting between the two halves of the clean glass U-tubes. This apparatus was sealed using PTFE tape (12 m x 12 mm x 0.075 mm) and the U-tube arms clamped in position, and 1 mL water added to each arm to check for leaks and to keep the membrane hydrated. The water was removed, and 3 M solutions of $CaCl_2 \cdot 2H_2O$ and $(NH_4)_2SO_4$ were passed through 0.22 µm syringe filters, and 1 mL of each was added to the individual arms of the U-tube. The membrane was isolated after 1-16 hours and rinsed with water. Any surface crystals were removed by scraping with a glass cover slip, and any unmineralized areas of the membrane were trimmed off. The polycarbonate was then dissolved in 1.8 mL dichloromethane (DCM) in an Eppendorf tube and sonicated for 30 seconds. 200 µL water was floated on top of the DCM, and the tube vortexed to transfer the sample to the water-DCM interface. The DCM was removed and replaced 2 times, and then removed *via* pipette, leaving the sample stored in water.

Bulk Ethanolic Precipitation of Bassanite

This was carried out according to the method of Tritschler *et al.* (2015)[1] where a 50 mM solution of $CaCl_2 \cdot 2H_2O$ and a 50 mM solution of $(NH_4)_2SO_4$ were prepared and passed through a 0.22 µm filter. 2.5 mL of each solution was combined for 1 second before pouring the mixture into 45 mL EtOH to quench the reaction. This was shaken vigorously for 30 seconds, then left to rest for 5 minutes. The mixture was centrifuged (5 minutes, 4,000 xg), the supernatant removed, and the precipitate washed by resuspension in 50 mL EtOH. The particles were stored in 5 mL EtOH.

Sample Characterization

Sample morphologies were determined using Scanning Electron Microscopy (SEM). Particles were resuspended in 5 µL of solvent (water for bulk gypsum and TE membrane samples, EtOH for bulk

bassanite samples) and applied to a 1 cm^2 piece of silicon wafer affixed to a SEM stub with copper tape. SEM images were recorded at 5 keV using an FEI Nova 450 NanoSEM using an in-lens secondary electron (SE) detector or a circular backscatter (CBS) detector, and energy dispersive X-ray (EDX) maps were recorded using a Bruker SDD-EDS detector at 18 keV. Transmission electron microscopy (TEM) was also used to characterize the particles, and in particular, to determine their structure using selected area electron diffraction (SAED). Samples were prepared by placing a 5 µL suspension of the prepared particles onto formvar (10 nm) and carbon (1 nm) coated copper grids (200 mesh) and allowed to dry. Analysis was carried out using a FEI Tecnai TF20: FEGTEM equipped with a Gatan Orius SC600A CCD camera operating at 200 keV using a spot size of 6 (electron dose ~30-50 $e^- Å^{-2}$ per image at 50,00 x magnification). Low dose TEM imaging and SAED patterns were recorded using an FEI Titan3 Themis 300: S/TEM with S-TWIN objective lens and monochromator (spread ~0.25 eV) operating at 300 keV and set to a screen current of 0.1-0.2 nA. This corresponded to an electron dose of ~2.5-5.0 $e^- Å^{-2}$ per image.

The structure of the calcium sulfate particles were also determined using powder X-ray diffraction (p-XRD) and Raman Spectroscopy. Sample in suspension were mixed with a silicon powder standard and dried onto a silicon substrate for p-XRD. Diffraction patterns were collected on a Brucker-AXS D8 series diffractometer using a Cu Kα source (40 kV, λ = 1.5406 Å) between 2θ = 2.0-50.0° (0.0196° and 3 seconds per step). These data were processed using Bruker-AXS Commander and EVA software, and the intensities normalized in OriginPro ver. 2018. Samples were dried onto silicon wafer to collect Raman spectra on a Horiba LabRAM HR Evolution Raman microscope using a green 532 nm 50 W laser at 5-10% power between 50-500 and 550-1200 cm^{-1}. The space in the spectrum collection between 500-550 cm^{-1} was selected to omit a very strong peak at 520 cm^{-1} from the silicon wafer substrate, and so avoid saturating the detector. The spectra were collected using LabSpec 6 software, with a spectral resolution of 0.2 cm^{-1} obtained using an 1800 grooves mm^{-1} grating and a 100 µm aperture, and plotted and normalized in OriginPro ver 2018.

Images were processed using Gatan Microscopy Suite Digital Micrograph version 3.30.2016.0 and / or Fiji[2, 3] version 1.151n_x64-x86 of imageJ2. Diffraction standards were obtained from the American Mineralogist Crystal Structure Database (AMCSD),[4] namely #4651 for gypsum, #6909 for bassanite, #5117 for anhydrite, and #15108 for silicon (Supplementary Table S2). These *d* spacings were converted to .cmdf files using CrystalMaker® ver. 9.2.9f1, and fitted to single crystal SAED patterns using SingleCrystal™ ver 2.3.3.

Computational Studies

The potential model of Byrne *et al.* (2017)[5] was used to produce the free energies of water transport in bassanite. The structure of bulk bassanite was built based on AMCSD #6909.[6] {001} and {110} surfaces were generated using the METADISE code.[7] Slabs were ≈30 Å thick, and hydrated with water occupying approximately 30 Å on either side. MD simulations were performed using the LAMMPS code[8] with a 1 fs time step. Slab calculations used the dipole correction of Ballenegger *et al*. (2009).[9]

Lattice equilibration was performed in an NPT ensemble employing a Nosé-Hoover thermostat set to 300 K and barostat set to 0 bar[10, 11] with relaxation times of 0.1 and 1.0 ps respectively. All lattice vectors in bulk bassanite were allowed to vary, whereas in the slab calculations, only the lattice vectors in the plane of the slab were allowed to vary. The systems were allowed to relax under the target conditions for 100 ps before the lattice vectors were averaged every 100 fs for 500 ps. Before the PMF calculations were performed, the lattice vectors were fixed at their NPT average. A water molecule was inserted/removed as required for the mechanism under study and the lattice vectors were assumed not to change from the fully hydrated value.

The Potential Mean Force (PMF) calculations were performed by constraining an atom in a position using a harmonic well and recording the average force the well applies during an MD simulation.[12] The average force exerted by the harmonic well is exactly opposite that being applied by the rest of the system on the atom of interest. The integral of the average force as a function of position gives the free energy profile associated with the pathway. All our PMF calculations used a spring constant of 10.0 eV $Å^{-2}$ applied in one direction only. All PMF calculations were performed in an NVT ensemble using a Langevin thermostat[13] with a 0.1 ps relaxation time. Forces were averaged every 10 fs. Slab calculations were simulated for 1 ns to allow thorough exploration of the 2D surface. Bulk calculations were simulated for 100 ps. Integration of the average force to obtain the free energy profiles was performed using the trapezoidal rule.

To analyze the flux to a growing crystal, the advection-diffusion equation was solved using COMSOL Multiphysics (ver. 5.5). This was done for (1) confinement within pores by using two large reservoirs connected by a narrow cylindrical channel with a crystal positioned at its center and (2) for a bulk solution using a crystal in the middle of a large reservoir. A concentration of 1 Mol m^{-3} and 0 Mol m^{-3} were assigned to the top and bottom of the large reservoirs (Figure 9a). Initially, the concentration at the surface of the crystal was also set to 0 Mol m^{-3}. A diffusion coefficient of 1×10^{-9} m^2 s^{-1} was used throughout and was taken as representative of the diffusion coefficients of 0.79×10^{-9} $m^2 s^{-1}$ for Ca^{2+} and 1.10×10^{-9} $m^2 s^{-1}$ for SO_4^{2-},[14] and the properties of the fluid taken as water. The boundary conditions represent a sink for the transported ions, under the limit of fast growth. In practice this is a simplification of the crystallization process, nevertheless measuring the diffusive flux at the crystal surface allows the relative transport of ions to be compared, and the importance (or not) of the geometry to be elucidated.

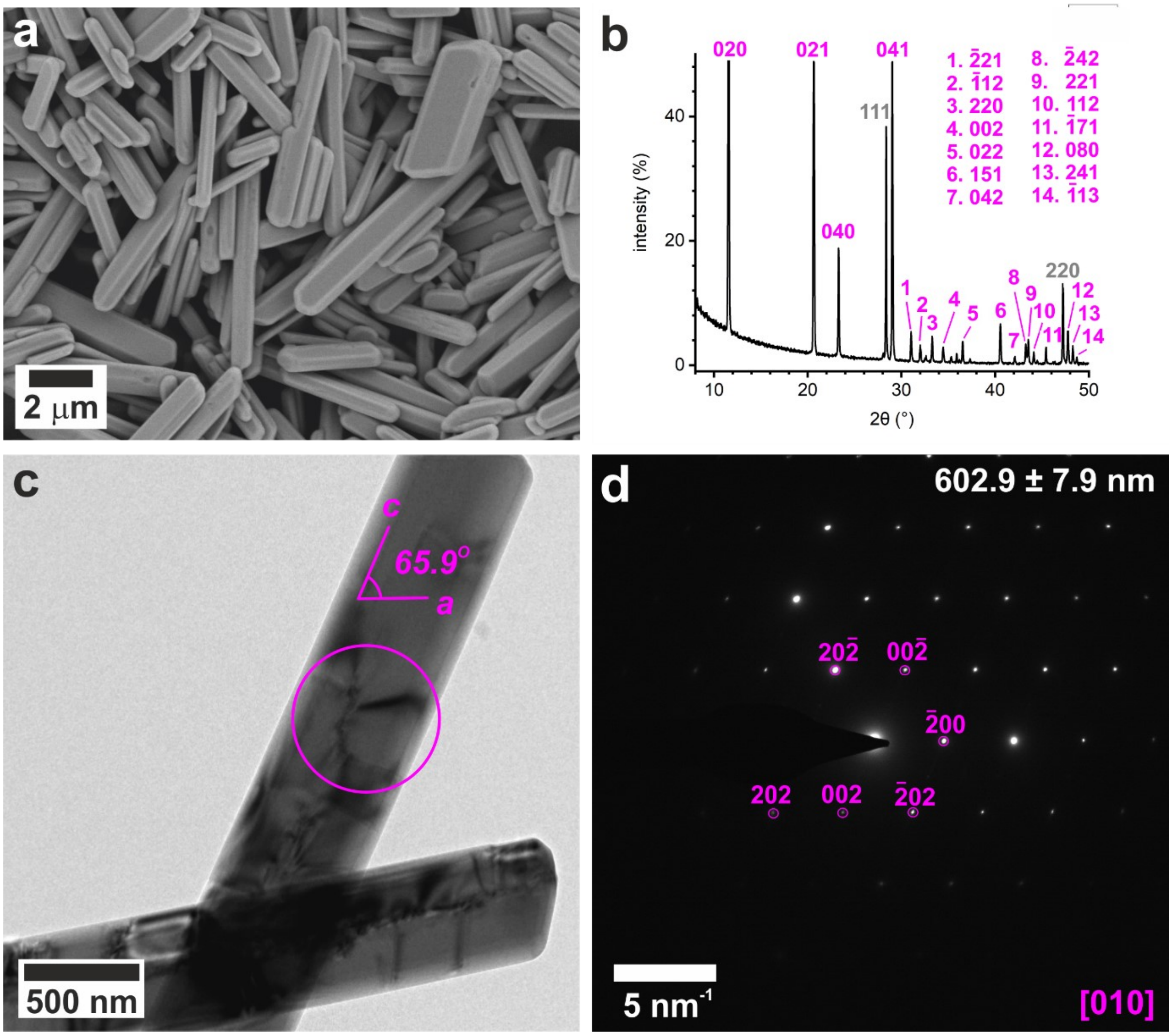

Supplementary Figure S1. Gypsum crystals from a bulk solution. (a) SEM image, (b) p-XRD pattern, (c) TEM image, and (d) corresponding SAED pattern. Circle in (c) indicates area selected for diffraction in (d), which is annotated with the crystal width and the fit to gypsum [010].

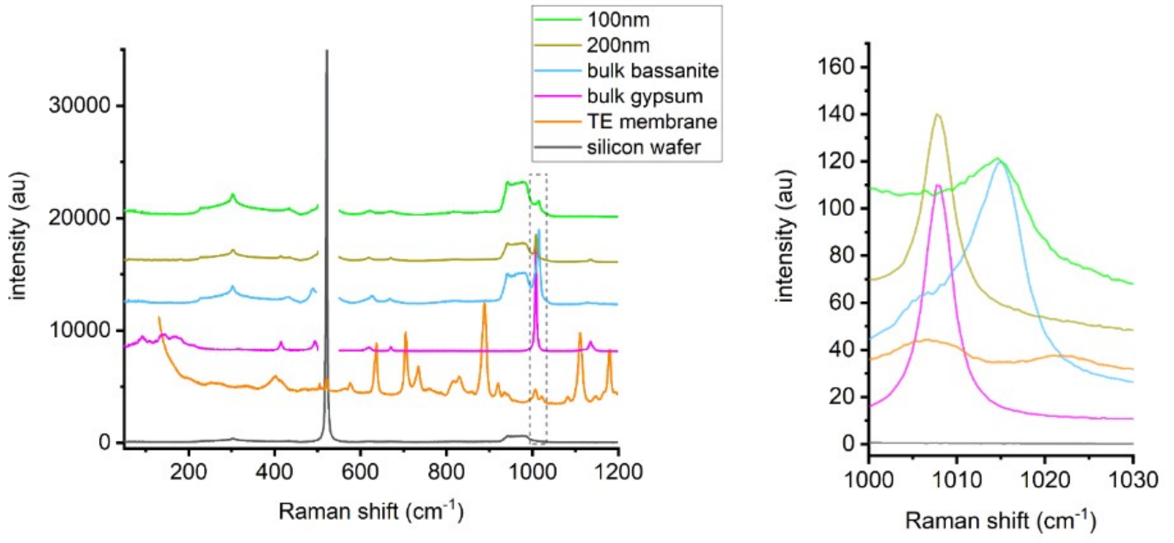

Supplementary Figure S2. Raman spectra of calcium sulfate precipitates. Left shows as collected and right shows normalized zoomed area to highlight shifts in v_1 symmetric sulfate peak for gypsum at 1008 cm^{-1} to bassanite at 1015 cm^{-1} in Raman spectra.[15, 16] Blank silicon wafer substrate (grey), TE membrane (orange), bulk gypsum precipitate (magenta), bulk ethanolic bassanite control (cyan), rods from 200 nm diameter pores (dark yellow), and rods from 100 nm diameter pores (green). It was not possible to collect spectra from the samples formed in the smaller diameter pores as their signal was too low.

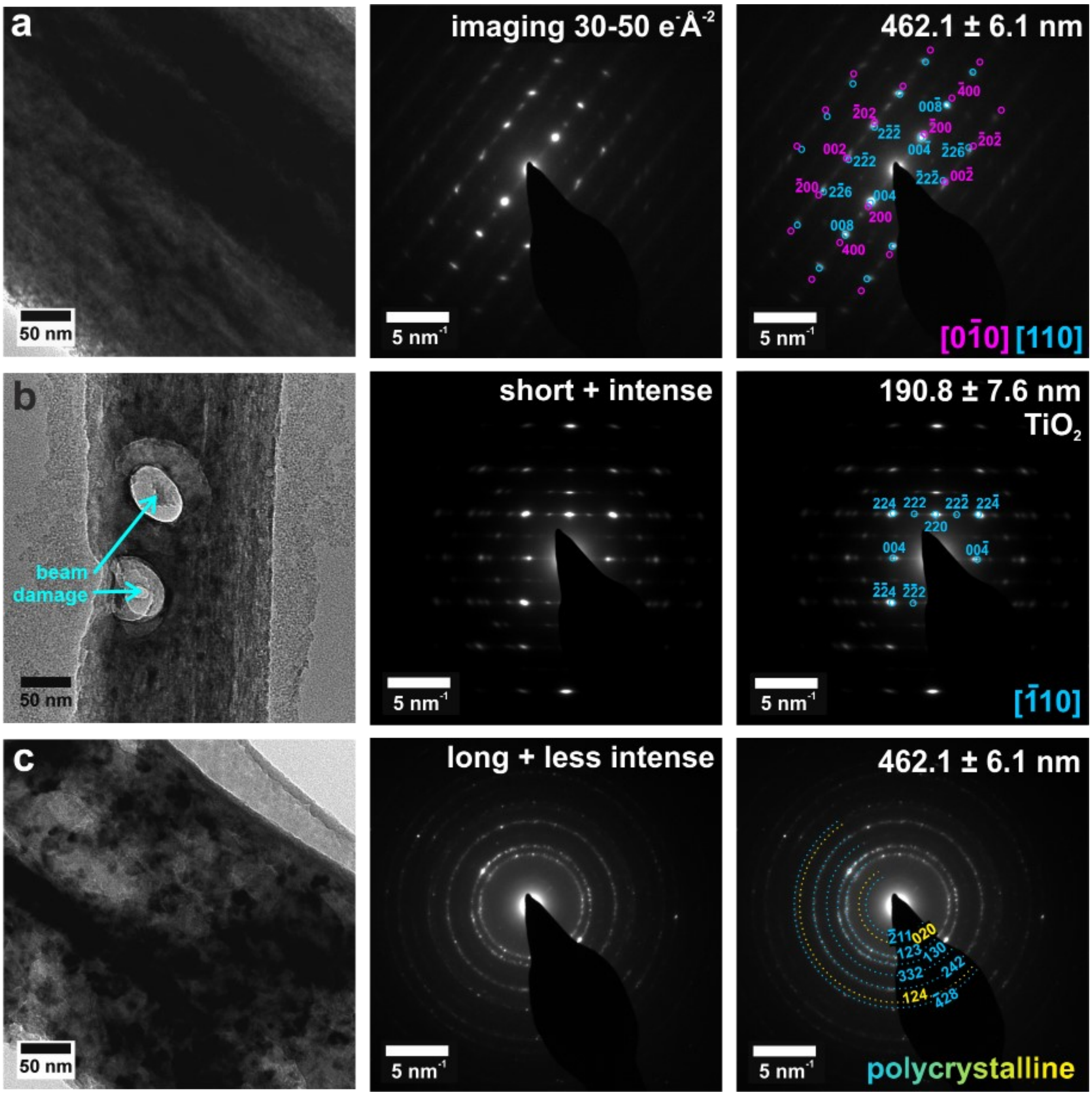

Supplementary Figure S3. TEM and SAED of beam damaged and control gypsum samples. TEM images (left), circle shows area selected for diffraction, and an unannotated (center) and annotated (right) diffraction pattern. (a) 30-50 e-Å^{-2} s^{-1} SAED does not fit to any gypsum or bassanite crystals. (b) Short intense beam exposure recrystallizes to bassanite. (c) Prolonged exposure shows polycrystalline bassanite and anhydrite.

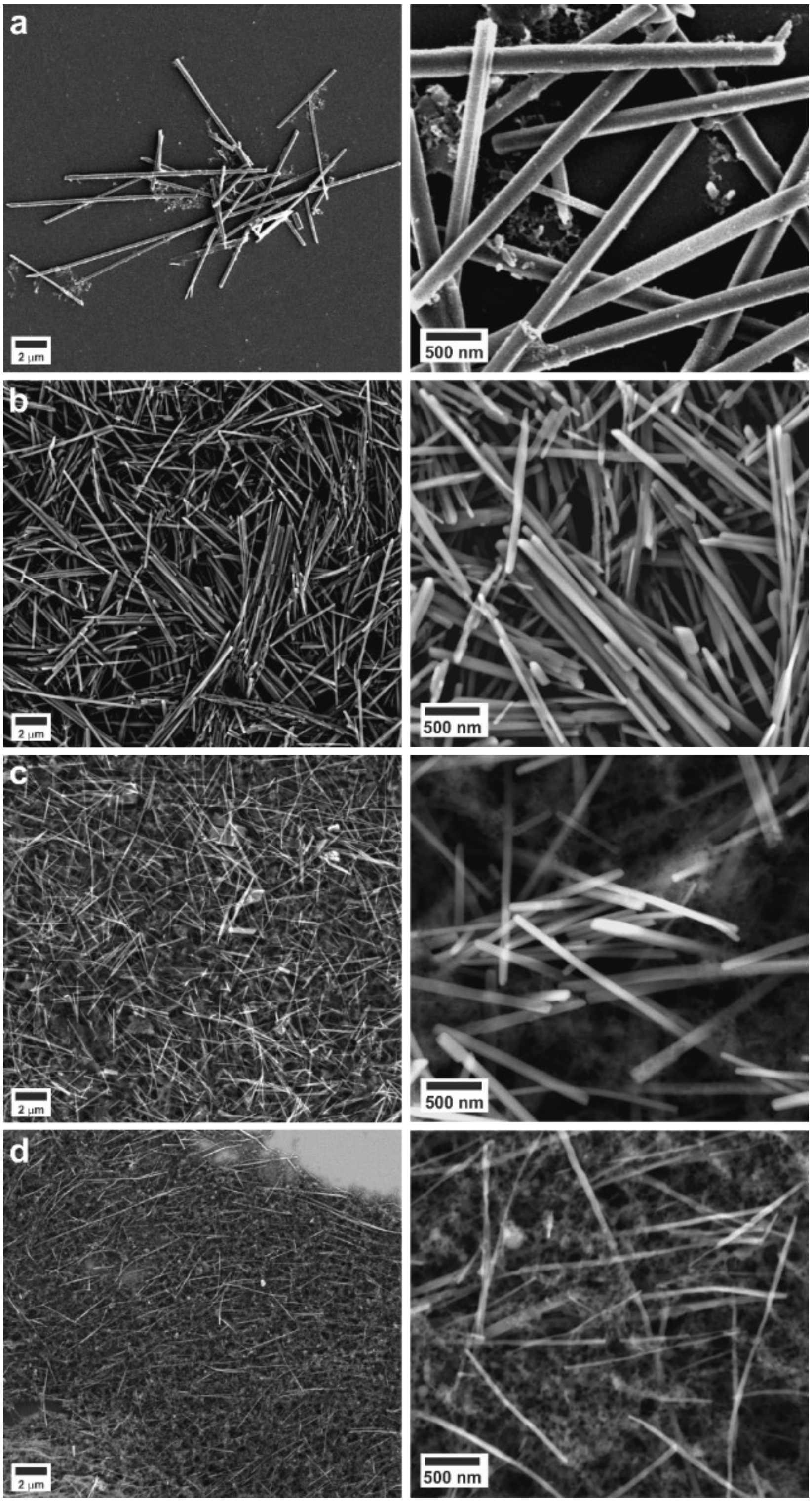

Supplementary Figure S4. SEM images of calcium sulfate rods formed in TE membrane pores. Images at low (left) and higher (right) magnification of calcium sulfate formed in pores of diameter (a) 200 nm, (b) 100 nm, (c) 50 nm, and (d) 25 nm. 16 hours mineralization time, some electron light material surrounding the rods is likely to be residue from membrane dissolution.

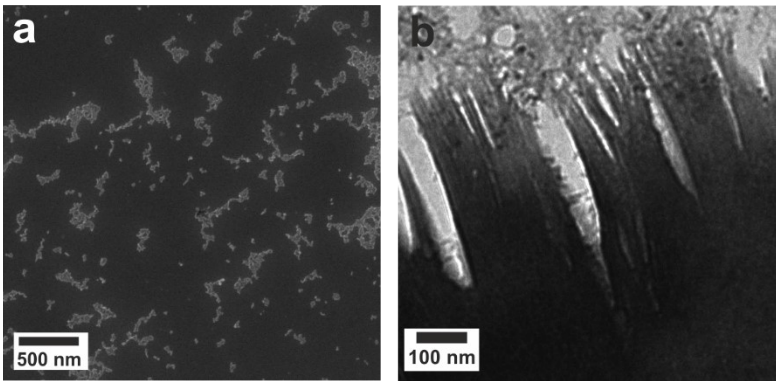

Supplementary Figure S5. SEM and TEM of calcium sulfate crystallized within 10 nm pores. (a) SEM image of disaggregated nanoparticles recovered from non-TiO_2 coated 10 nm pores. TEM images at of samples formed within TiO_2 coated 10 nm TE membrane pores (measured 8.8 ± 0.2 nm).

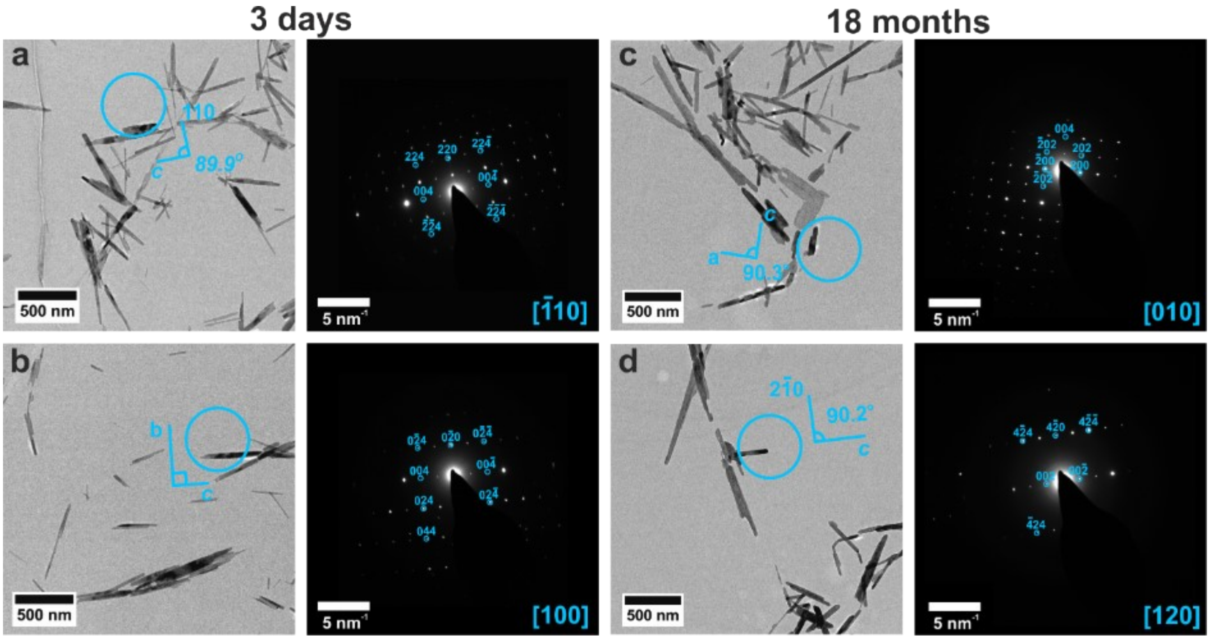

Supplementary Figure S6. TEM and SAED of bulk ethanolic bassanite, formed as described in Tritscheler *et al.* (2015)[1]. Examples of bassanite rods precipitated in ethanol and their corresponding SAED patterns (a & b) imaged within 3 days of synthesis and (c & d) at 18 months post-synthesis. The long axis of the crystal rod was aligned with the *c*-axis of bassanite, whereas the short axes align in (a) to the [110], in (b) to the *b* axis [010], in (c) to the *a* axis [100], and in (d) to the [2$\bar{1}$0].

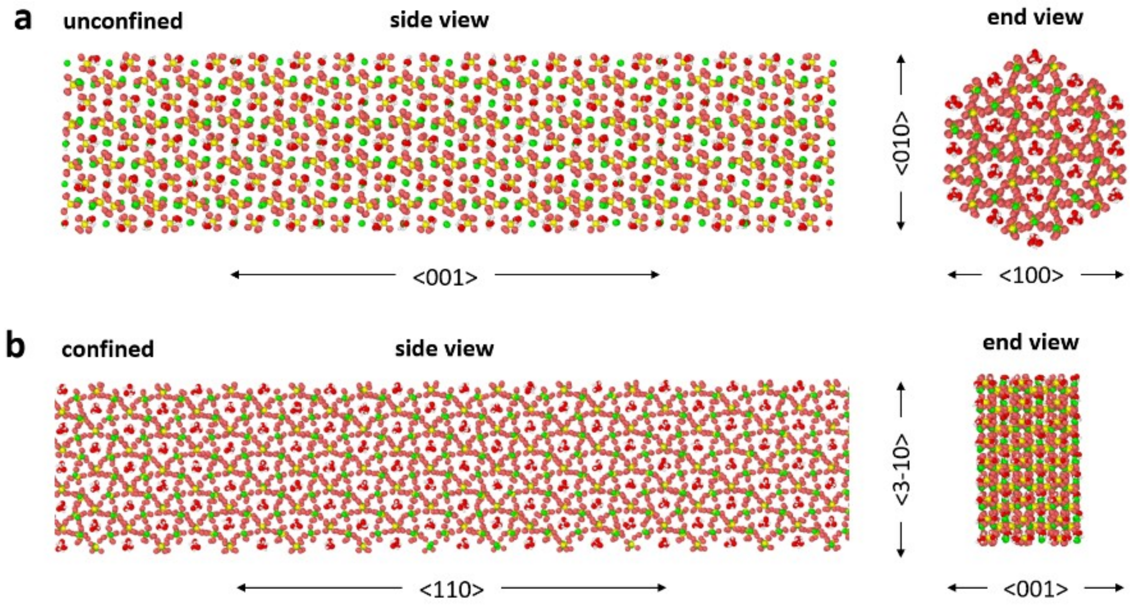

Supplementary Figure S7. Diagram of major crystallographic orientations for bulk and confinement templated bassanite. In the nanorods precipitated from the (a) bulk, the ends of the water channels, which are open on the (001) face and run the full length of the long axis of the rod, so are minimally expressed on the small end faces of the rods. In the (b) confinement templated bassanite, the open ends of the water channels run perpendicular to the long axis, so will appear all along the length of the rod.

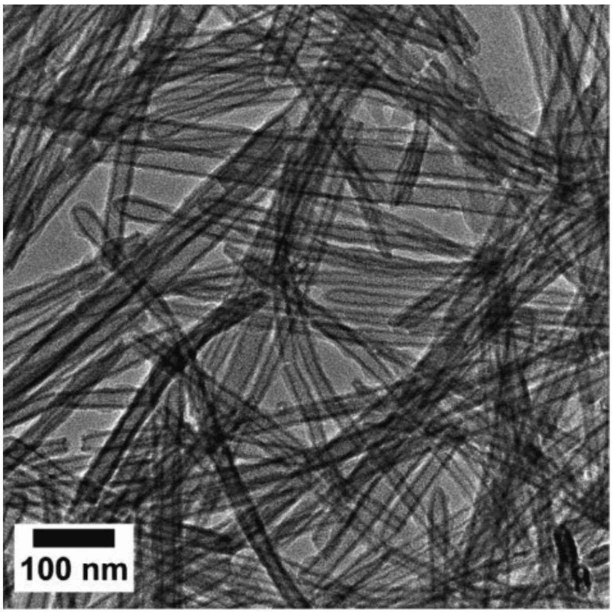

Supplementary Figure S8. TEM of TiO_2 deposited using 200 cycles of ALD on 25 nm TE pore membranes. Many of the tube ends were closed off when 200 cycles of ALD are used to coat the smallest pores, which means that they cannot be used for crystallization in confinement, as the reactants would be unable to get into the interior of the structures. As such, 100 cycles of ALD were used to deposit a thinner layer of TiO_2 on the 10 nm and 25 nm pores to make more open-ended TiO_2 tubes available for mineralization.

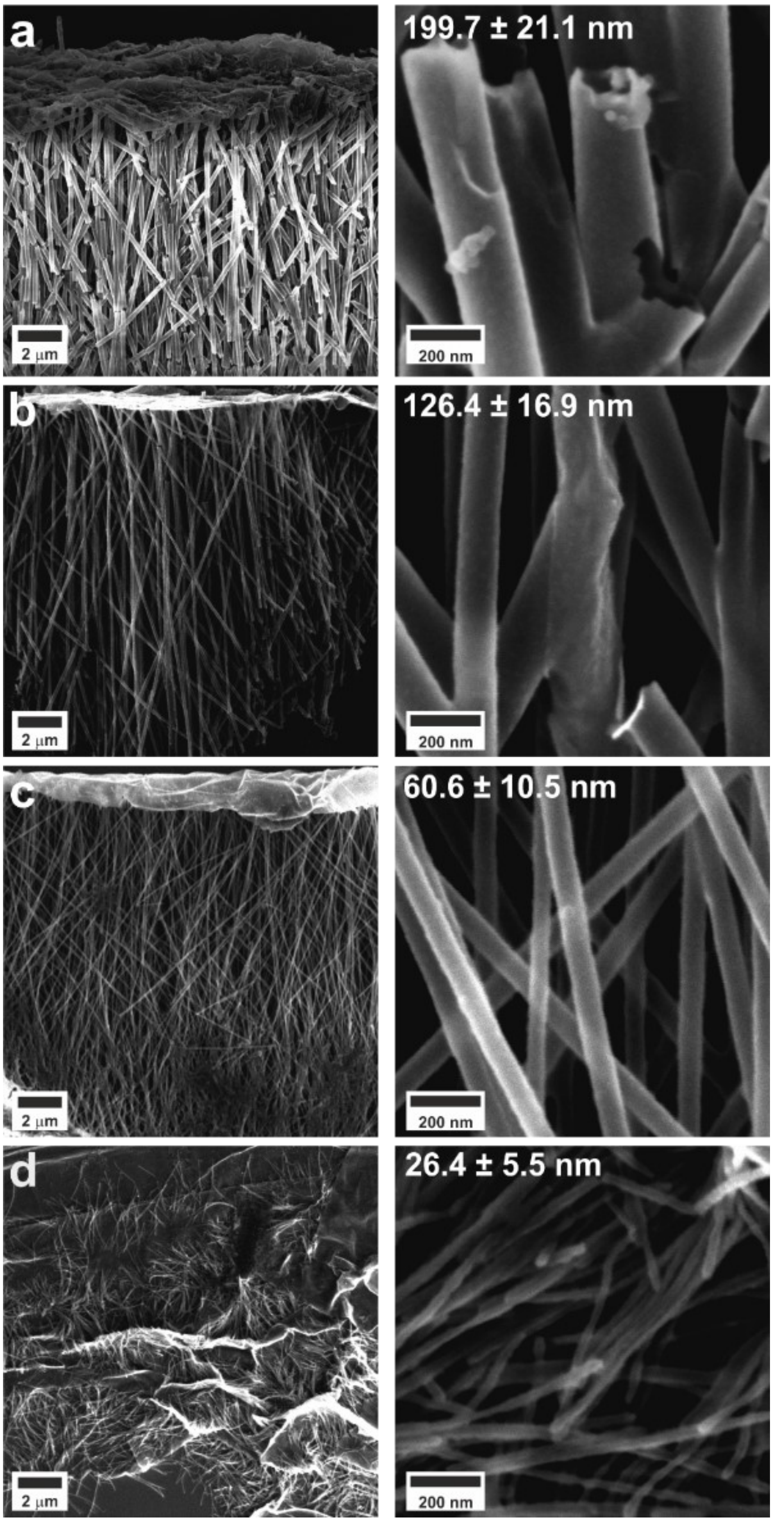

Supplementary Figure S9. SEM of TiO_2 tubes after membrane removal. SEM images show two different magnifications of tubes formed in TE membranes with a manufacturer quoted diameter of (a) 200 nm, (b) 100 nm, (c) 50 nm, and (d) 25 nm. These are annotated with the external diameter of the TiO_2 tubes measured in imageJ, summarized in Table S3. The tubes formed from the smallest 10 nm pores could not be imaged in SEM.

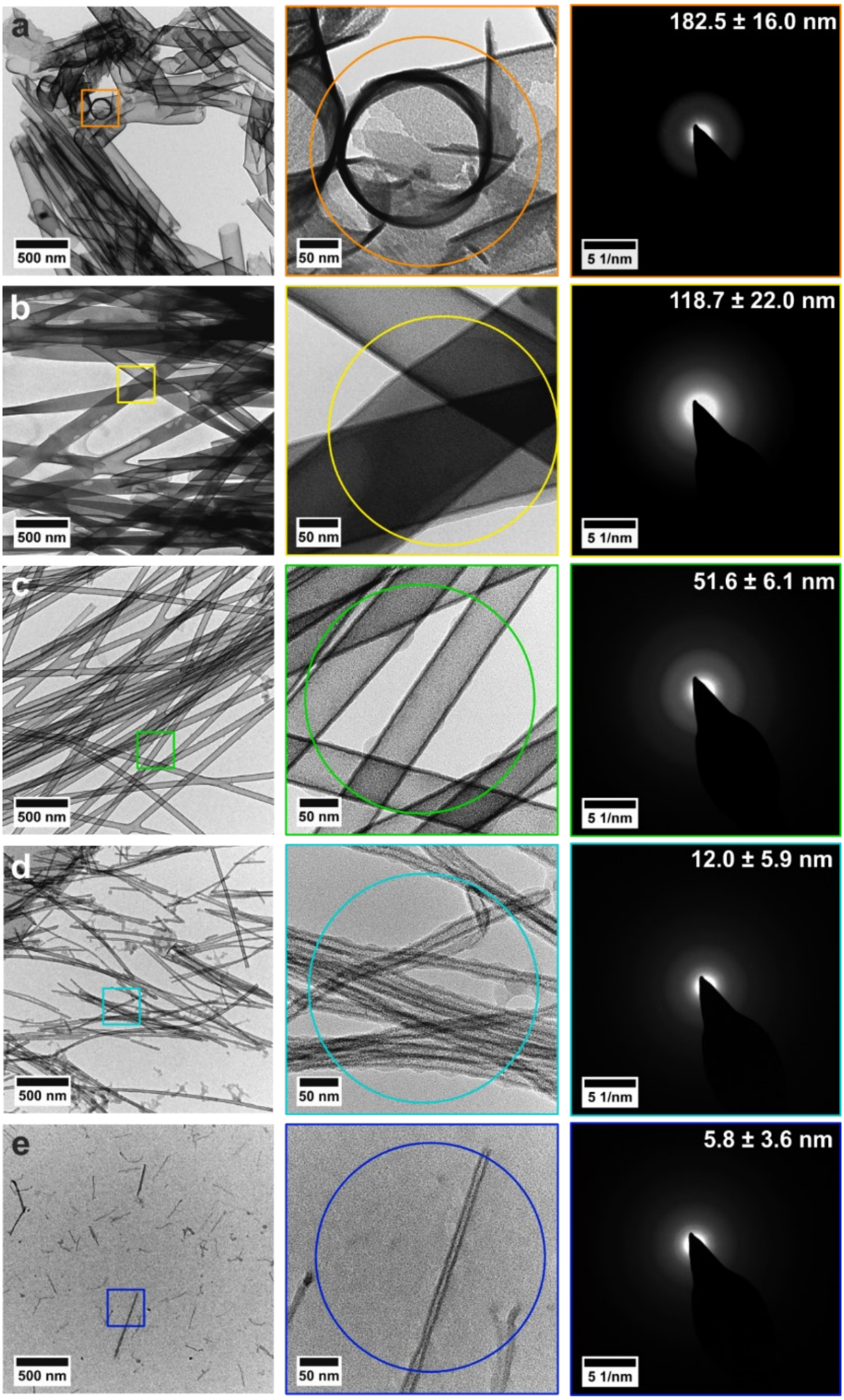

Supplementary Figure S10. TEM and SAED of TiO_2 tubes. TEM images show two different magnifications, right shows SAED. These are from pores with the manufacturer quoted pore diameter of (a) 200 nm, (b) 100 nm, (c) 50 nm, (d) 25 nm, and (e) 10 nm, and are annotated with the internal diameter of the TiO_2 tubes measured in imageJ, summarized in Supplementary Table S1.

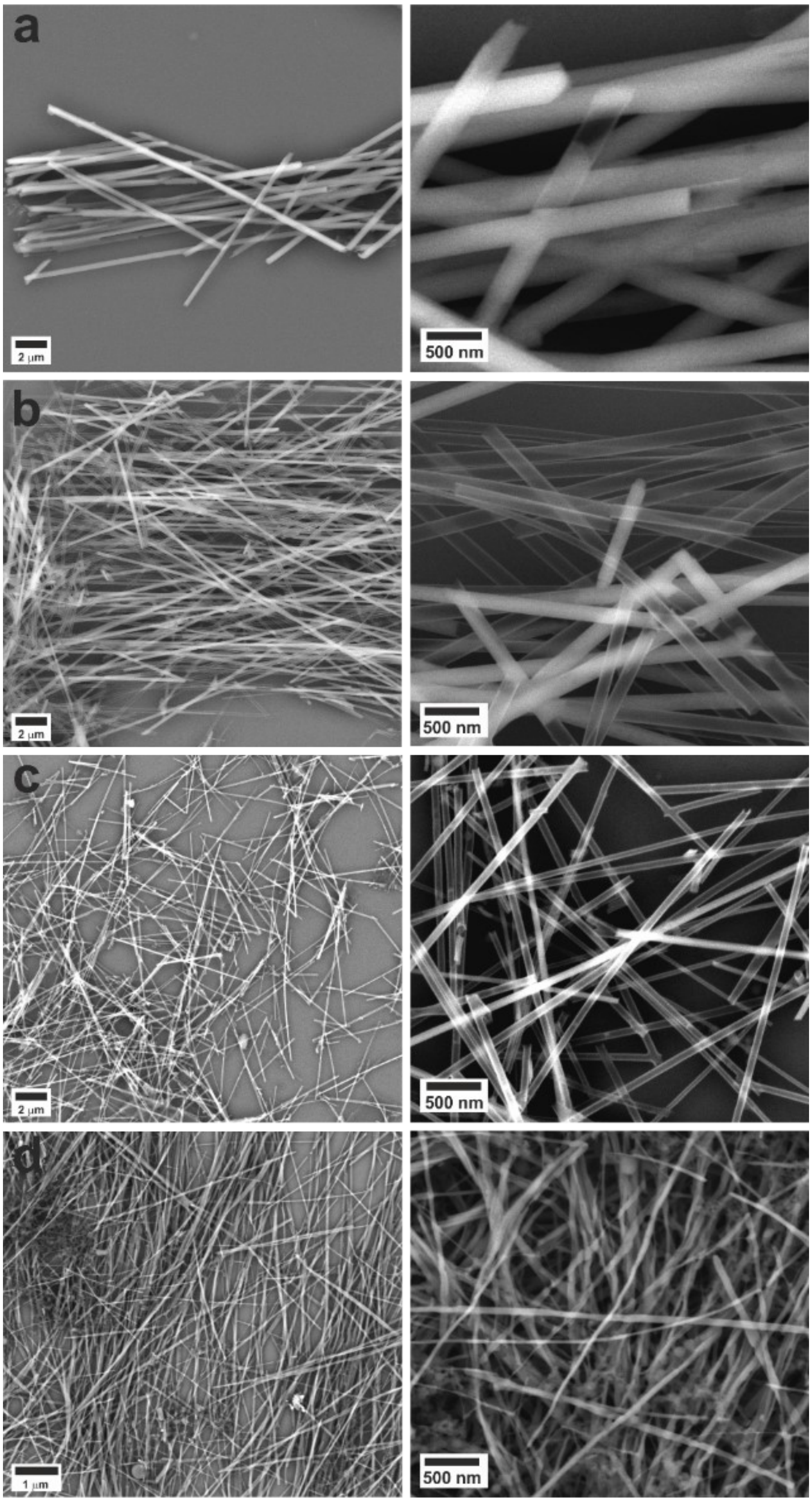

Supplementary Figure S11. SEM of calcium sulfate rods mineralized in TiO_2 coated TE membrane pores. Images of samples at low (left) and high (right) magnification from manufacturer specified pore diameters of (a) 200 nm, (b) 100 nm, (c) 50 nm, and (d) 25 nm TiO_2 coated TE pores after 16 hours mineralization. Filled tubes show up a white on the images as they contain more electron dense materials than the empty areas. It was not possible to image the 10 nm filled tubes using SEM.

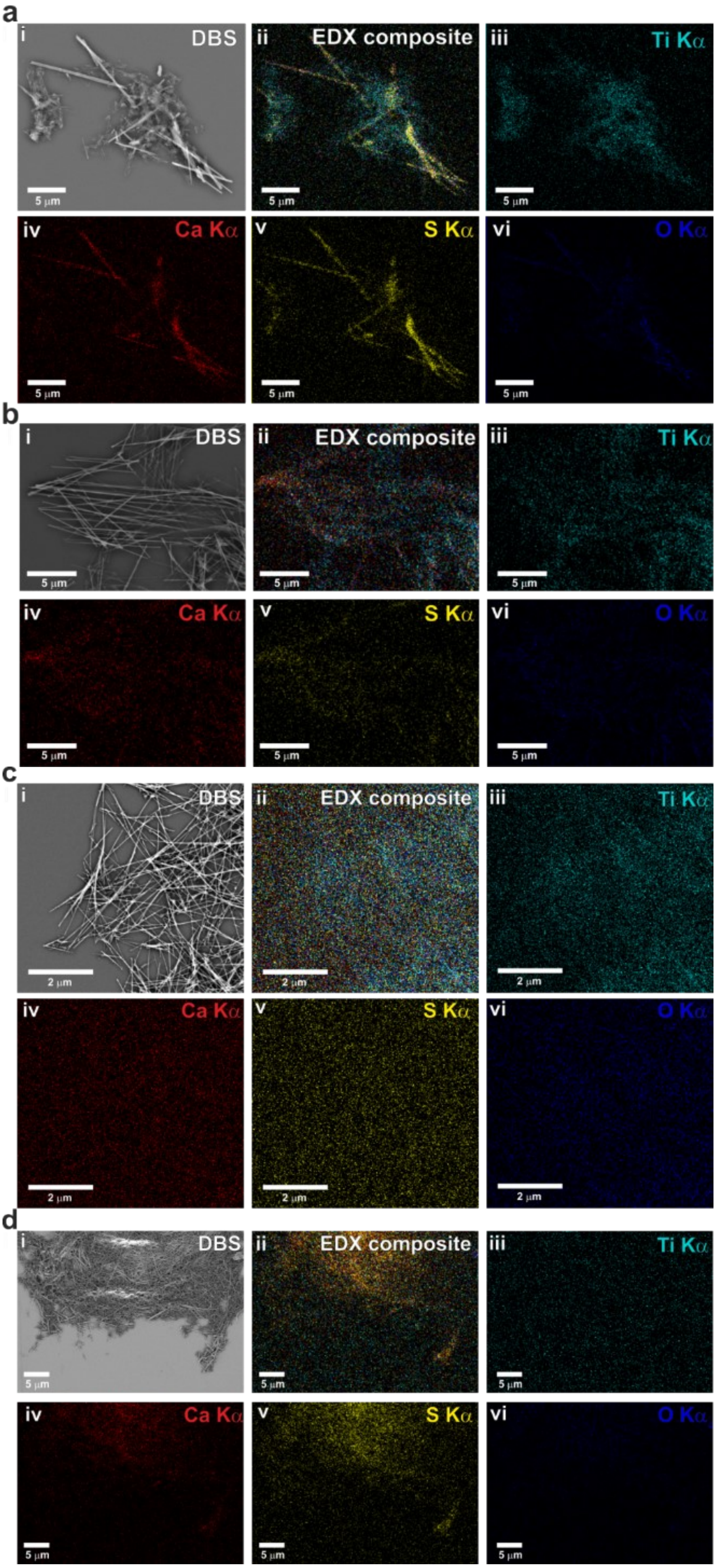
a
i
DBS
5 μm
ii
EDX composite
5 μm
iii
Ti Kα
5 μm
iv
Ca Kα
5 μm
v
S Kα
5 μm
vi
O Kα
5 μm
b
i
DBS
5 μm
ii
EDX composite
5 μm
iii
Ti Kα
5 μm
iv
Ca Kα
5 μm
v
S Kα
5 μm
vi
O Kα
5 μm
c
i
DBS
2 μm
ii
EDX composite
2 μm
iii
Ti Kα
2 μm
iv
Ca Kα
2 μm
v
S Kα
2 μm
vi
O Kα
2 μm
d
i
DBS
5 μm
ii
EDX composite
5 μm
iii
Ti Kα
5 μm
iv
Ca Kα
5 μm
v
S Kα
5 μm
vi
O Kα
5 μm

Supplementary Figure S12. SEM and EDX of TiO_2 tubes mineralized with calcium sulfate. Images in (i) show SEM image, (ii) shows a composite elemental map of the area imaged in (i); with individual maps of (iii) Ti Kα in cyan, (iv) Ca Kα in red, (v) S Kα in yellow, and (vi) O Kα in blue. The interaction volume for X-rays is much larger than for secondary electrons,[17] it is not possible to resolve individual mineralized rods within the smaller (100 – 25 nm) tubes individually, but it is possible to see that calcium and sulfur are localized to groups of mineralized tubes. Suite (a) is of calcium sulfate deposited within 200 nm, (b) is 100 nm, (c) in 50 nm, and (d) is 25 nm, TiO_2 tubes at 16 hours mineralization.

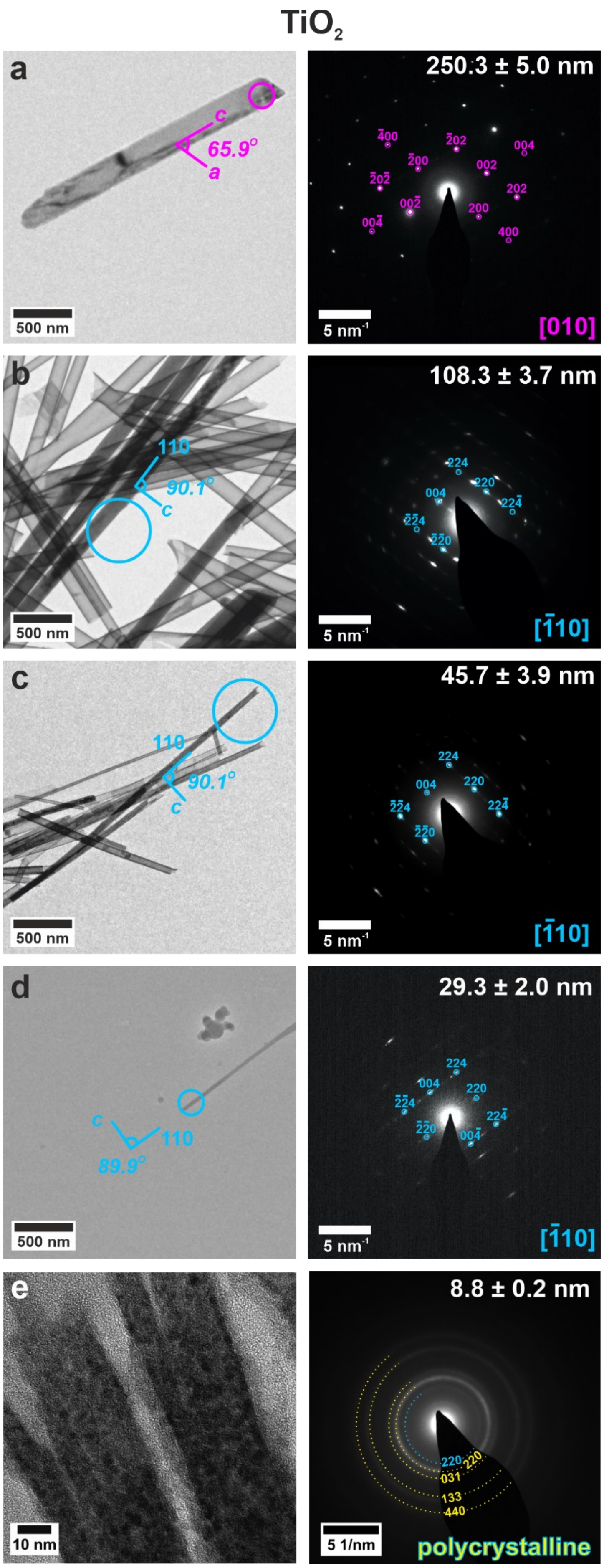
TiO2
a
c
65.9°
a
500 nm
250.3 ± 5.0 nm
5 nm-1
[010]
b
110
90.1°
c
500 nm
108.3 ± 3.7 nm
5 nm-1
[1̄10]
c
110
90.1°
c
500 nm
45.7 ± 3.9 nm
5 nm-1
[1̄10]
d
c
110
89.9°
500 nm
29.3 ± 2.0 nm
5 nm-1
[1̄10]
e
10 nm
8.8 ± 0.2 nm
5 1/nm
polycrystalline

Supplementary Figure S13. TEM images (left) and SAED patterns (right) of calcium sulfate precipitated within titania nanotubes with pore diameters of (a) 200 nm, (b) 100 nm, (c) 50 nm, (d) 25 nm and (e) 10 nm. TEM images (left) are annotated with a circle indicating the area selected for diffraction. Gypsum reflections are shown in pink, bassanite in cyan and anhydrite in yellow. Measured crystal diameters are annotated on SAED patterns.

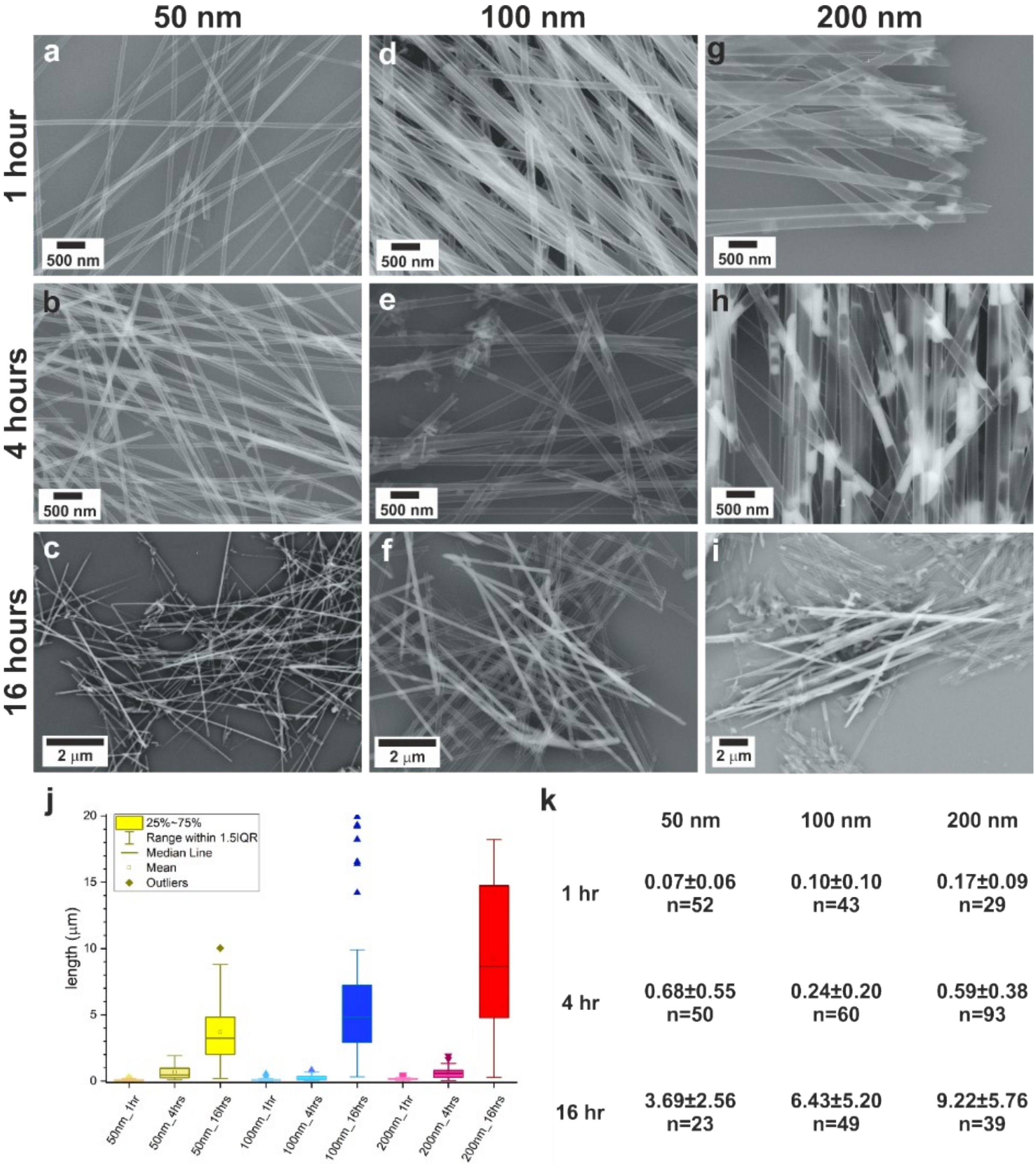

Supplementary Figure S14. SEM images of calcium sulfate formed within TiO_2 coated TE membrane pores at 1 hr (a, d & g), 4 hr (b, e & h) and 16 hr (c, f & i) mineralization. Samples from (a-c) 50 nm, (d-f) 100 nm, and (g-i) 200 nm TiO_2 coated TE membrane pores. (j) Summary plot of crystal rod length within tubes and (k) average length ± standard deviation in μm and n number of crystals measured.

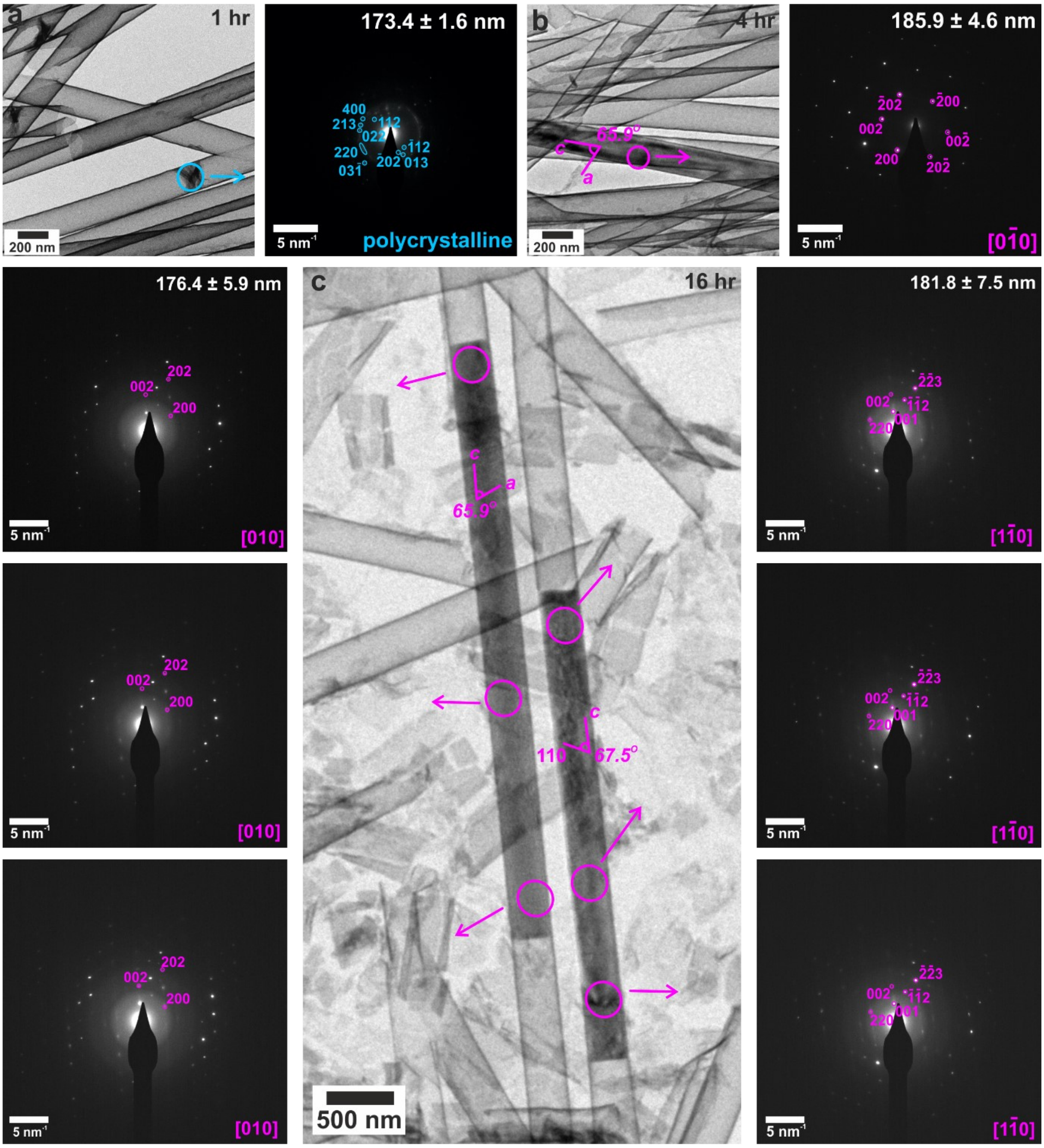

Supplementary Figure S15. TEM images and corresponding SAED patterns of calcium sulfate precipitated within 200 nm diameter (manufacturer quoted) TiO_2 nanotubes after (a) 1 h, (b) 4 h and (c) 16 h. Area selected for diffraction is indicated by circles on TEM images, arrow indicates corresponding SAED pattern. Bassanite reflections are labelled in cyan and gypsum in pink, and the latter consistently have the *c* axis aligned parallel to the long axis of the crystals.

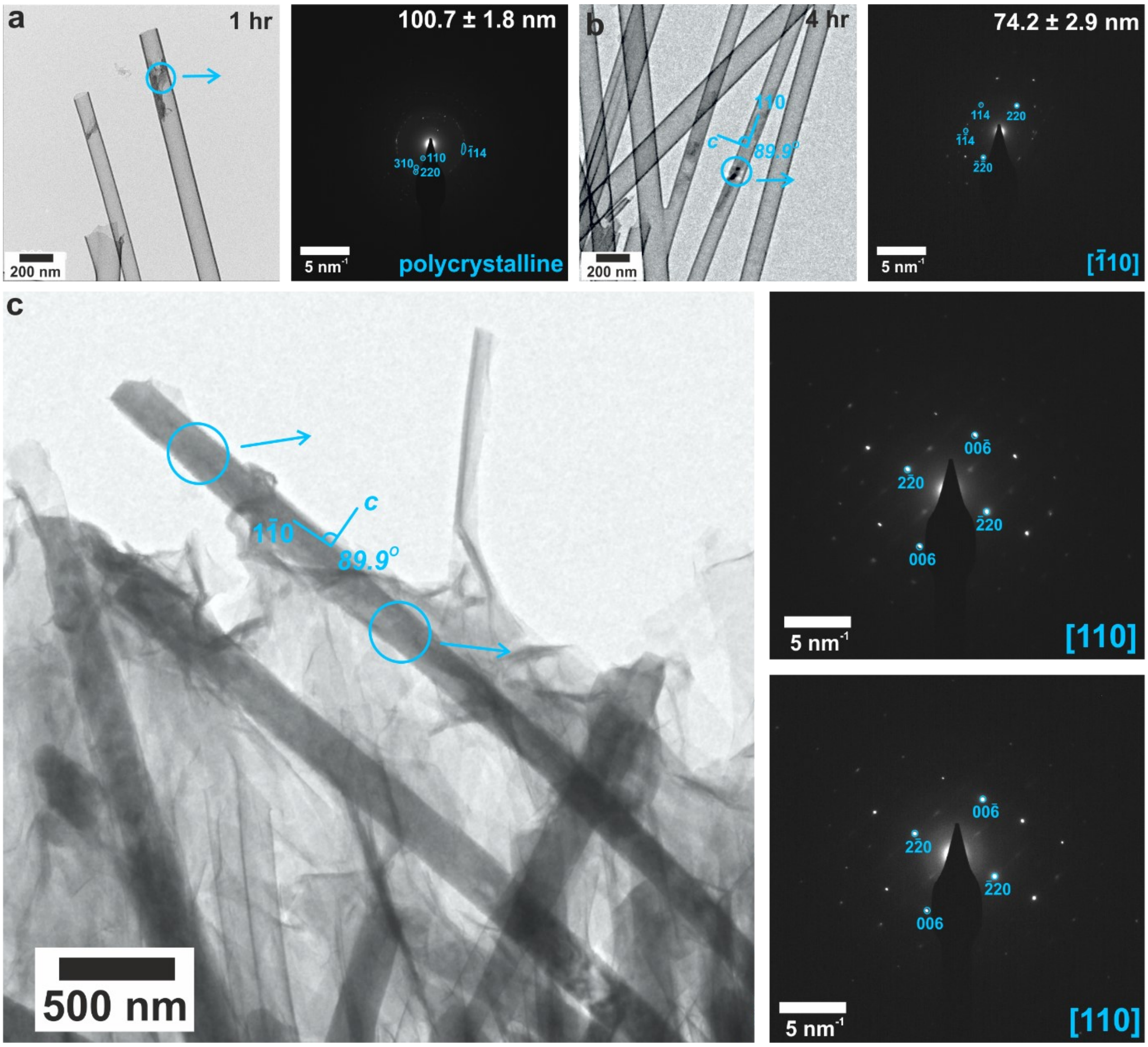

Supplementary Figure S16. TEM and SAED of calcium sulfate crystallized within TiO_2 coated 100 nm TE membrane pores after 1-16 hours mineralization. Samples from (a) 1 hr, (b) 4 hr, and (c) 16 hr. Bassanite reflections labelled in cyan. Small polycrystalline material was observed at 1 hour (a), small aligned crystals at 4 hours (b), and long single aligned crystal rods at 16 hours (c).

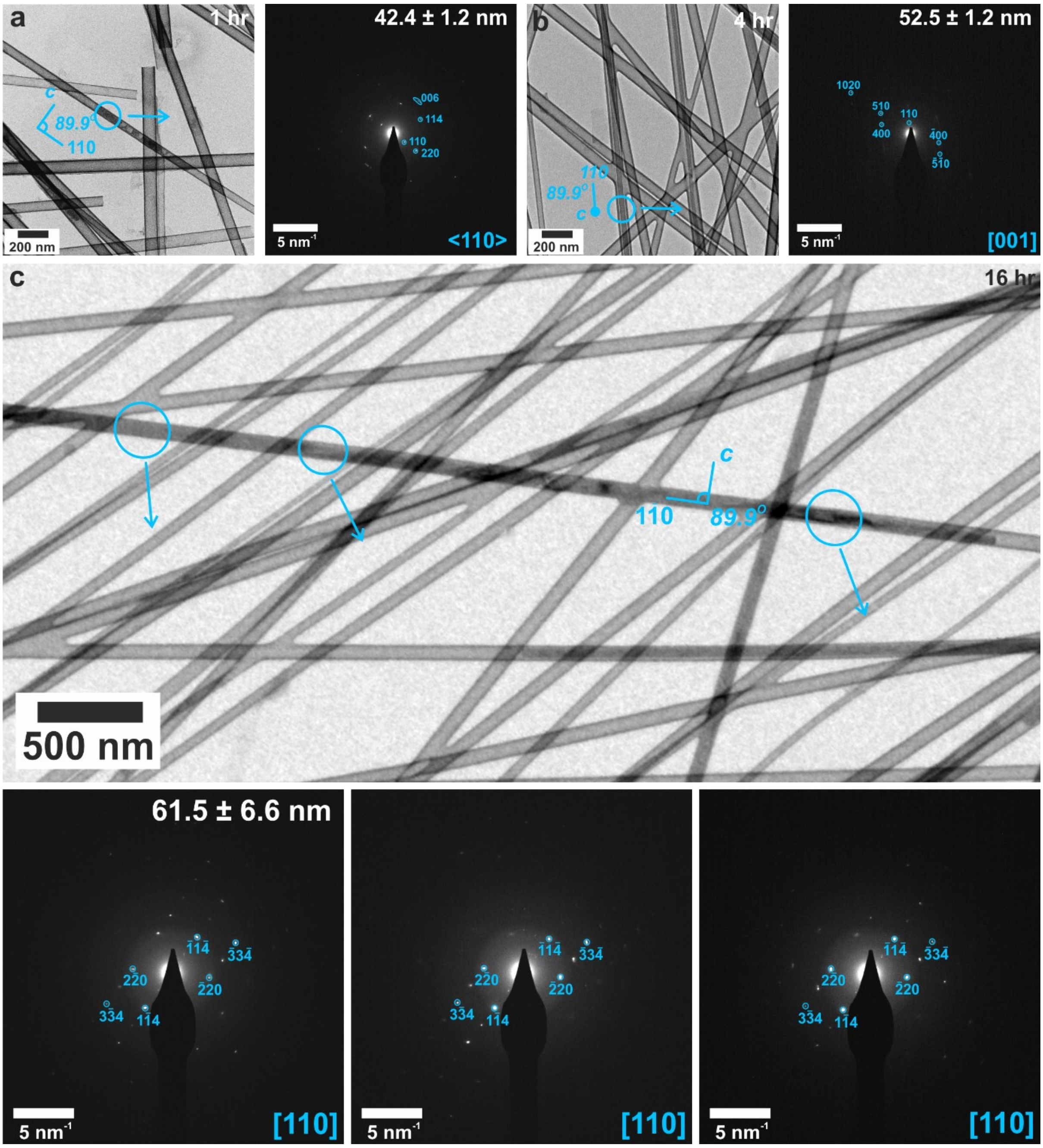

Supplementary Figure S17. TEM and SAED of calcium sulfate crystallized within TiO_2 coated 50 nm TE membrane pores after 1-16 hours mineralization. Samples from (a) 1 hr, (b) 4 hr, and (c) 16 hr. Bassanite reflections labelled in cyan. Small, partially aligned polycrystalline material was observed at 1 hour (a), small aligned crystals at 4 hours (b), and long single aligned crystal rods at 16 hours (c).

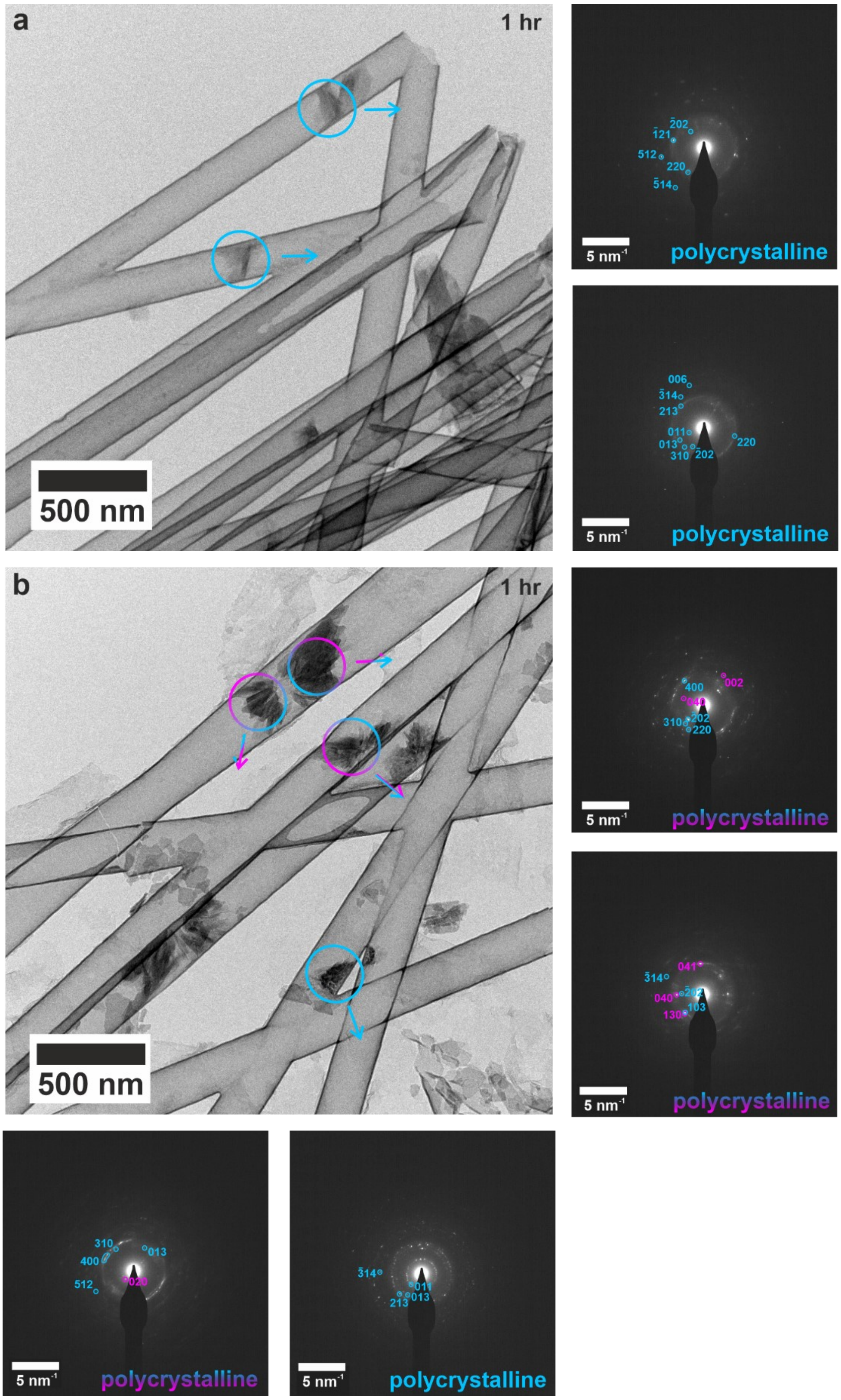

Supplementary Figure S18. TEM and SAED of 200 nm diameter tubes after 1 hour mineralization. Area selected for diffraction is indicated by circles on the TEM images, arrows point to location of corresponding SAED pattern. Bassanite reflections are labelled in cyan and gypsum in pink. (a) All small mineral plugs index to polycrystalline bassanite, and (b) slightly larger mineral plugs from same time-point show both bassanite and gypsum reflections.

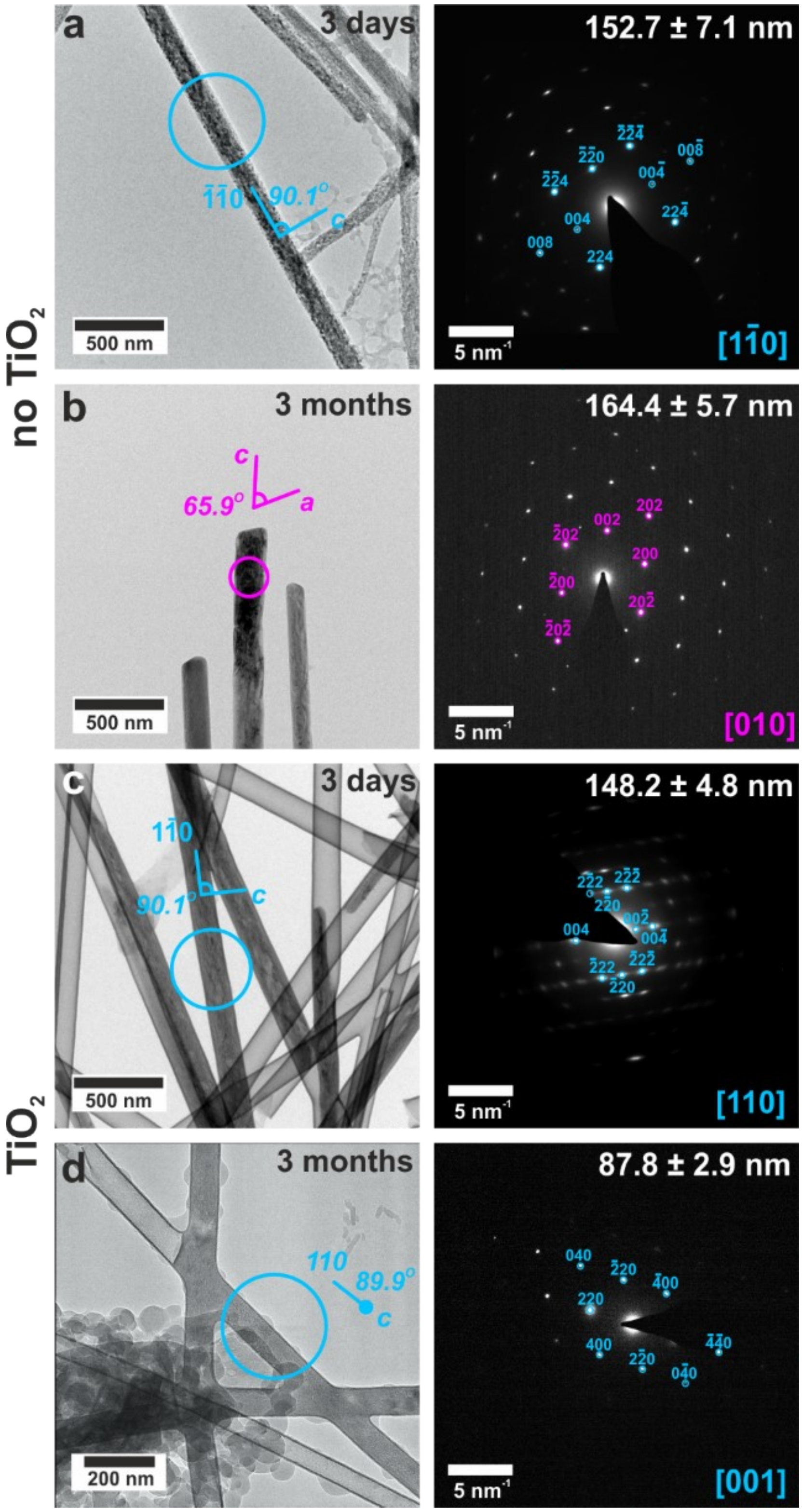

Supplementary Figure S19. TEM and SAED of calcium sulfate crystallized within uncoated and TiO_2 coated 100 nm (manufacturer quoted) TE membrane pores before and after aging for 3 months in air. Calcium sulfate precipitated in 100 nm uncoated TE membrane pores indexes initially (a) as bassanite, whereas after 3 months (b) this fits to gypsum. Within the TiO_2 coated TE membrane pores, the calcium sulfate indexed as bassanite both (c) initially and (d) at 3 months.

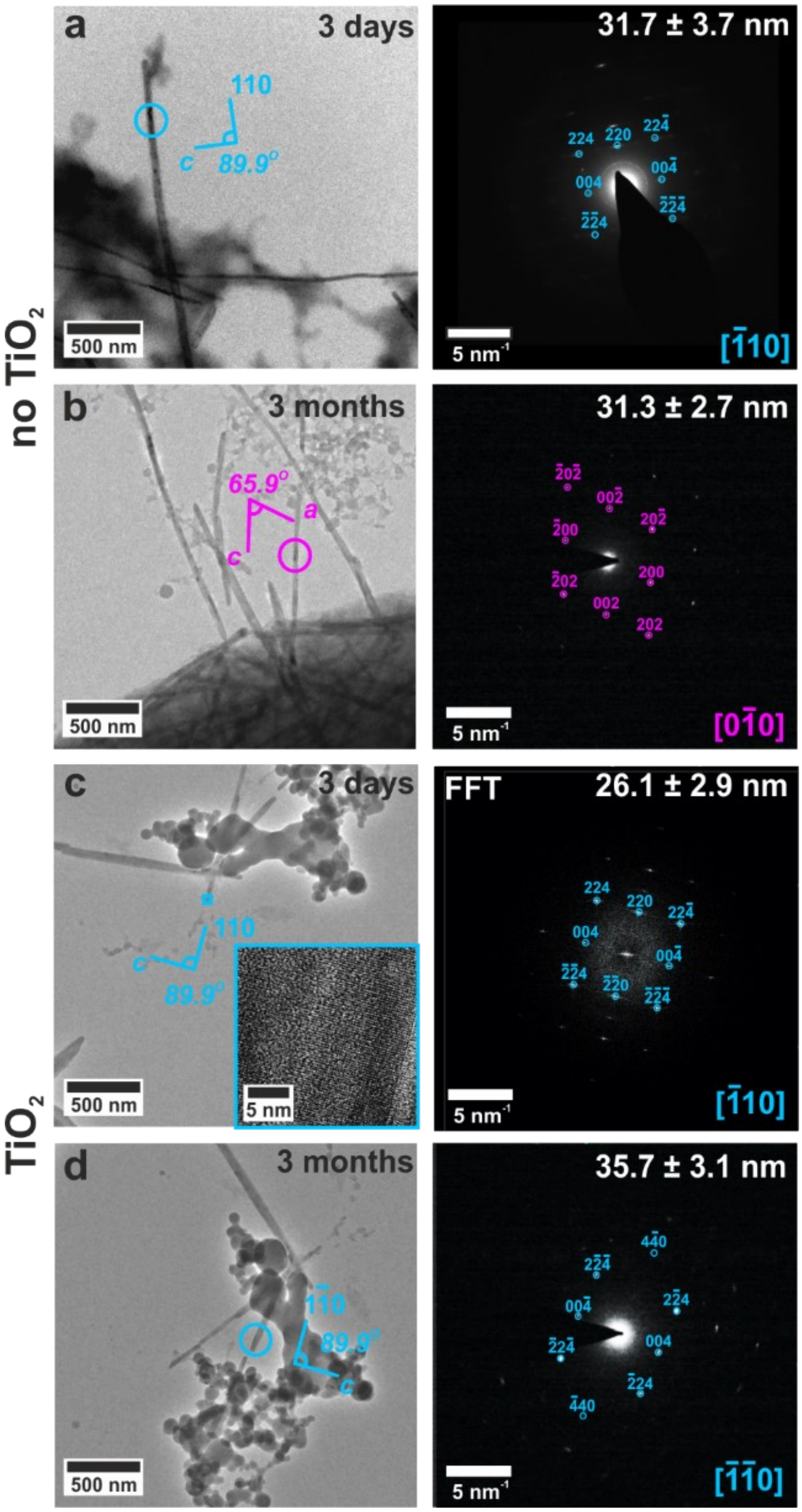

Supplementary Figure S20. TEM images and corresponding SAED patterns of calcium sulfate precipitated within 25 nm (manufacturer quoted) TE membrane pores before and after aging for 3 months in air. Area selected for diffraction is circled on the TEM image, gypsum reflections labelled in pink and bassanite in cyan. In the absence of a titania nanotube, the template crystals are (a) bassanite, but (b) transform to gypsum within 3 months. In the presence of a titania coating (a) the original bassanite crystals (b) remain unchanged after 3 months.

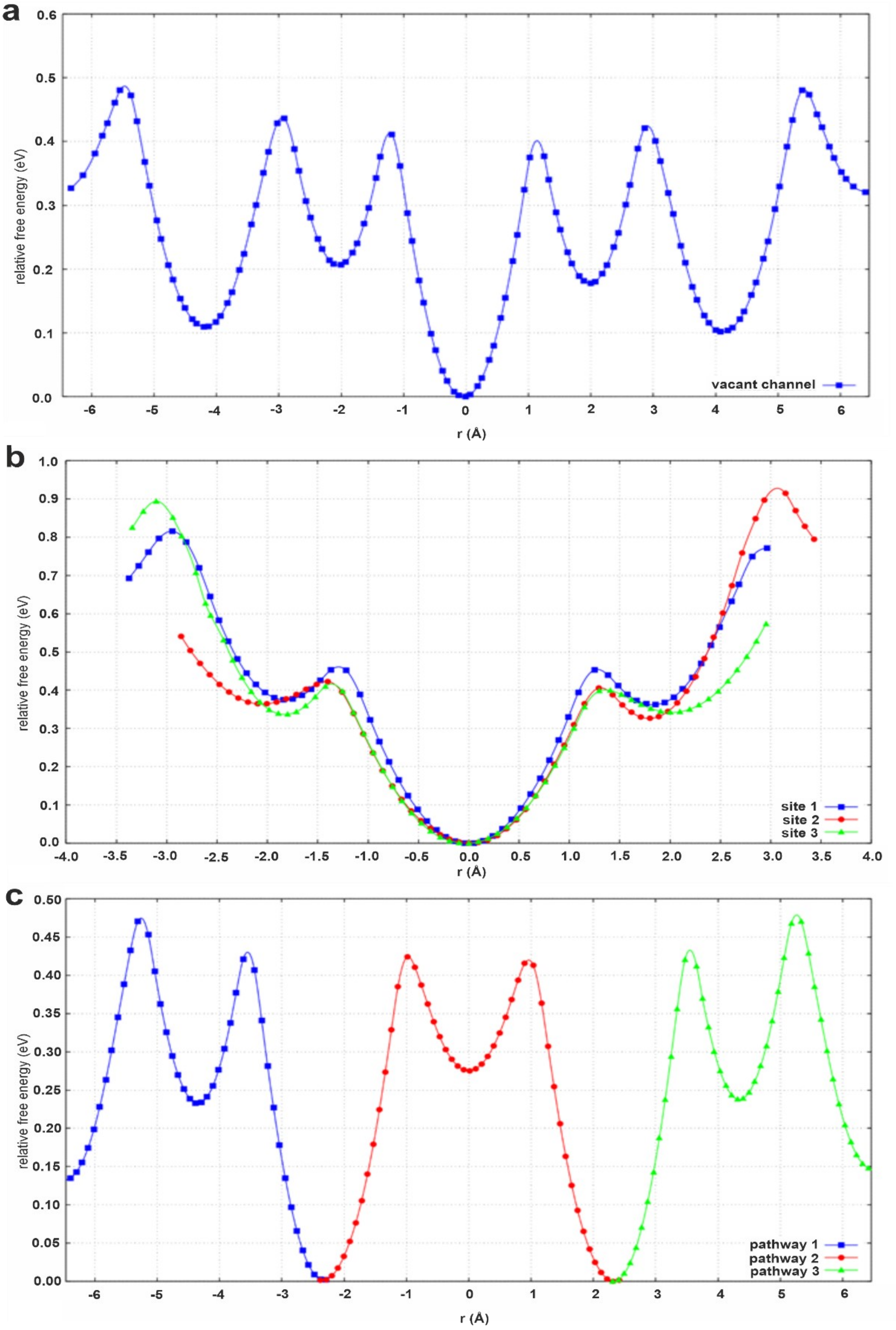

Supplementary Figure S21. Graphs of energy barriers for water and / or vacancy migration through bassanite water channels. (a) Migration of a single water molecule through an otherwise vacant bassanite water channel. (b) Free energy barriers to creating a water Frenkel pair in an occupied bassanite water channel. Each water site is plotted showing the self-symmetry at site 1, and that sites 2 and 3 are mirror images of each other. (c) Free energy barriers to migration of a water vacancy through an occupied bassanite water channel. Each color represents a different vacancy migration pathway and are truncated where the pathways overlap, showing this is a fully periodic pathway.

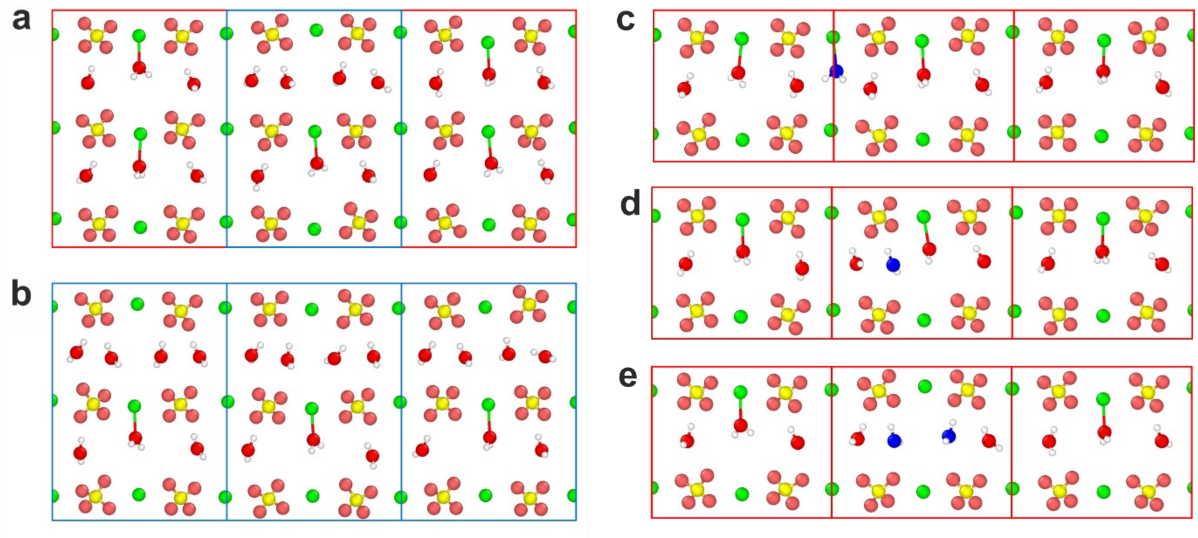

Supplementary Figure S22. Snapshots of water in bassanite structures. (a) Slice through two adjacent water channels in regular bassanite *i.e.* $CaSO_4.0.5H_2O$ (red boxed areas) with water molecules arranged corresponding to the minimum at +/- 3.23 Å in the blue central box, which is more like that observed in $CaSO_4.0.625H_2O$. (b) Same view as shown in (a), but entire pore length is $CaSO_4.0.625H_2O$, showing water ordering in this more hydrated bassanite structure. Water molecule arrangement in pore at (c) the global minimum (+/- 0 Å), (d) the second minimum (+/- 1.74 Å), and (e) the third minimum (+/- 3.23 Å) of the plot shown in Figure 7d.

#	action	volume	time
1	flow nitrogen	50 sccm	
2	flow argon	150 sccm	
3	wait		30 seconds
4	Set APC	0 %	
5	pulse TDMAT		1 second
6	wait		1 second
7	flow nitrogen	20 sccm	
8	flow argon	60 sccm	
9	wait		120 seconds
10	set APC	7 %	
11	wait		15 seconds
12	flow nitrogen	5 sccm	
13	flow argon	5 sccm	
14	set APC	100 %	
15	wait		120 seconds
16	set APC	0 %	
17	pulse water		0.64 seconds
18	wait		1 second
19	flow nitrogen	20 sccm	
20	flow argon	60 sccm	
21	wait		120 seconds
22	set APC	7 %	
23	wait		15 seconds
24	flow nitrogen	5 sccm	
25	flow argon	5 sccm	
26	set APC	100 %	
27	wait		90 seconds
28	go to 3 for 100-200 cycles		

Supplementary Table S1. Optimized recipe for TiO_2 deposition within TE membrane pores. Where sccm is standard cubic centimeters per minute, and APC is automatic performance control valve, each cycle takes ≈8 minutes 35 seconds.

name / AMSCD #		a (Å) / α (°)	b (Å) / β (°)	c (Å) / γ (°)
gypsum		6.2770	15.1810	5.6720
I2/a	4651	90.000	114.110	90.000
0.5 bassanite		12.035	6.9294	12.6705
I2	6909	90.000	90.266	90.000
0.625 bassanite		13.869	13.869	12.7189
P3_221	19834	90.000	90.000	120.000
anhydrite		6.9930	6.9950	6.2450
Amma	5117	90.000	90.000	90.000
silicon		5.448	5.448	5.448
Fd3m	15108	90.000	90.000	90.000

Supplementary Table S2. Unit cell parameters from AMSCD database entries for calcium sulfate polymorphs used in this study. AMCSD for gypsum # 4651,[18] 0.5 bassanite # 6909,[6] 0.625 bassanite # 19834,[19] and anhydrite # 5117.[20] Silicon (AMSCD #15108)[21] is also shown as silicon powder standard was used to align diffraction patterns.

pore diameter (nm)	No. ALD cycles	TEM internal diameter (nm)	SEM external diameter (nm)	TEM TiO_2 thickness (nm)
200	200	182.5 ± 16.0	199.7 ± 21.1	8.4 ± 1.3
100	200	118.7 ± 22.0	126.4 ± 16.9	10.2 ± 0.4
50	200	51.6 ± 6.1	60.6 ± 10.5	6.6 ± 0.9
25	100	12.0 ± 5.9	26.4 ± 5.5	5.0 ± 0.4
10	100	5.8 ± 3.6	n/a	4.3 ± 1.2

Supplementary Table S3. Measurements of unmineralized TiO_2 tubes after membrane removal. The TE pore diameter is as quoted on the purchased TE membranes. ALD cycles refers to the number of atomic layer deposition cycles used to deposit the TiO_2 layer. The internal diameter and TiO_2 thicknesses were measured from TEM images, and the external diameter from SEM images. Measurements are an average of 50-150 measurements recorded in imageJ[2, 3], error quoted is 1 standard deviation of the average.

Condition	Polymorph	crystal description	alignment (long axis)	alignment (perpendicular axis)
200 nm TiO_2 pore 1 h	bassanite & gypsum	polycrystalline small plug	poor {110}	poor 001 / none
200 nm TiO_2 pore 4 h +	gypsum	single crystal elongated rods	001	010
100 nm TiO_2 pore 1 h	bassanite	polycrystalline small plug	poor {110}	none
100 nm TiO_2 pore 4 h +	bassanite	single crystal elongated rods	{110}	001
50 nm TiO_2 pore 1 h	bassanite	polycrystalline small plug	poor {110}	poor 001
50 nm TiO_2 pore 4 h +	bassanite	single crystal elongated rods	{110}	001
100-25 nm pore 3 days	bassanite	single crystal elongated rods	{110}	001
100-25 nm pore 3 months	gypsum	single crystal elongated rods	001	010
100-25 nm TiO_2 3 days	bassanite	single crystal elongated rods	{110}	001
100-25 nm TiO_2 3 months	bassanite	single crystal elongated rods	{110}	001

Supplementary Table S4. Summary of calcium sulfate crystallization in TE membrane pores when aged.

pore size (nm)	figure	TiO_2?	feature / age	diameter (nm)	± (nm)
bulk	S1	no	control	602.9	7.9
bulk*	S3a	no	30-50 $e^{-}Å^{2}$	462.1	6.1
200	S3b	yes	short intense	190.8	7.6
bulk*	S3c	no	longer	462.1	6.1
200	1e	no	16h	208.1	3.7
100	2a	no	16h	137.6	5.8
50	2b	no	16h	50.3	4.2
25	2c	no	16h	21.3	1.3
200	4a S13a	yes	16h	250.3	5.0
100	4b S13b	yes	16h	108.3	3.7
50	S13c	yes	16h	45.7	3.9
25	S13d	yes	16h	29.3	2.0
10	4c S13e	yes	16h	8.8	0.2
200	S15a	yes	1h	173.4	1.6
200	S15b	yes	4h	185.9	4.6
200	S15c L	yes	16h	176.4	5.9
200	S15c R	yes	16h	181.8	7.5
100	S16a	yes	1h	100.7	1.8
100	S16b	yes	4h	74.2	2.9
100+	S16c	yes	16h	obscured	
50	S17a	yes	1h	42.4	1.2
50	S17b	yes	4h	52.5	1.2
50	S17c	yes	16h	61.5	6.6
200	5a S18a	yes	1h	174.7	4.9
200	5a S18a	yes	1h	178.8	5.3
200	5b S18b	yes	1h	271.8	19.1
200	5b S18b	yes	1h	190.6	6.3
200	S18b	yes	1h	243.2	14.8
200	S18b	yes	1h	174.1	12.6
100	S19a	no	3 days	152.7	7.1
100	S19b	no	3 months	164.4	5.7
100	S19c	yes	3 days	148.2	4.8
100	S19d	yes	3 months	87.8	2.9
50	6a	no	3 days	52.7	2.1
50	6b	no	3 months	81.7	3.5
50	6c	yes	3 days	59.2	2.4
50	6d	yes	3 months	49.8	2.4
25	S20a	no	3 days	31.7	3.7
25	S20b	no	3 months	31.3	2.7
25	S20c	yes	3 days	26.1	2.9
25	S20d	yes	3 months	35.7	3.1

Supplementary Table S5. Calcium sulfate rod diameters quoted in this manuscript. TE membrane pores show a small variability about the diameter quoted as purchased. As such, measurements of the mineral rods that were used for diffraction were recorded from TEM images. 10 measurements of the rod diameter across the area selected for diffraction were recorded in imageJ2, 3, with the error quoted one standard deviation of these values.* Samples were from the same area but imaged after a minimal exposure of 30-50 $e\text{-}Å^{2}s^{-1}$ (S3a ≈1 sec) and after a longer exposure (S3c 2-3 min). + Edge of tube / rod unclear so not measured.

References

1. U. Tritschler, M. Kellermeier, C. Debus, A. Kempter and H. Cölfen, *CrystEngComm,* 2015, **17**, 3772-3776, DOI: 10.1039/C5CE00519A.
2. J. Schindelin, I. Arganda-Carreras, E. Frise, V. Kaynig, M. Longair, T. Pietzsch, S. Preibisch, C. Rueden, S. Saalfeld, B. Schmid, J.-Y. Tinevez, D. J. White, V. Hartenstein, K. Eliceiri, P. Tomancak and A. Cardona, *Nat. Methods,* 2012, **9**, 676-682, DOI: 10.1038/nmeth.2019.
3. J. Schindelin, C. T. Rueden, M. C. Hiner and K. W. Eliceiri, *Mol. Reprod. Dev.,* 2015, **82**, 518-529, DOI: 10.1002/mrd.22489.
4. M. Hall-Wallace and R. T. Downs, *Am. Mineral.,* 2003, **88**, 247-250, DOI: n/a.
5. E. H. Byrne, P. Raiteri and J. D. Gale, *J. Phys. Chem. C,* 2017, **121**, 25956-25966, DOI: 10.1021/acs.jpcc.7b09820.
6. P. Ballirano, A. Maras, S. Meloni and R. Caminiti, *Eur. J. Mineral.,* 2001, **13**, 985-993, DOI: 10.1127/0935-1221/2001/0013-0985.
7. G. W. Watson, E. T. Kelsey, N. H. de Leeuw, D. J. Harris and S. C. Parker, *J. Chem. Soc. Faraday Trans.,* 1996, **92**, 433-438, DOI: 10.1039/FT9969200433.
8. S. Plimpton, *J. Comp. Phys.,* 1995, **117**, 1-19, DOI: 10.1006/jcph.1995.1039.
9. V. Ballenegger, A. Arnold and J. J. Cerdà, *J. Chem. Phys.,* 2009, **131**, 094107, DOI: 10.1063/1.3216473.
10. S. Nosé, *J. Chem. Phys.,* 1984, **81**, 511-519, DOI: 10.1063/1.447334.
11. W. G. Hoover, *Phys. Rev. A,* 1985, **31**, 1695-1697, DOI: 10.1103/PhysRevA.31.1695.
12. A. R. Leach, *Molecular Modelling: Principles and Applications*, Pearson Education, London, U.K., 2001.
13. T. Schneider and E. Stoll, *Phys. Rev. B,* 1978, **17**, 1302-1322, DOI: 10.1103/PhysRevB.17.1302.
14. Aqion, Table of Diffusion Coefficients, https://www.aqion.de/site/diffusion-coefficients, (accessed Dec 5th, 2020).
15. Y. Liu, A. Wang and J. J. Freeman, presented in part at the 40th Lunar and Planetary Science Conference, The Woodlands, Texas, U.S.A., March 23-27, 2009.
16. N. Prieto-Taboada, O. Gómez-Laserna, I. Martínez-Arkarazo, M. Á. Olazabal and J. M. Madariaga, *Anal. Chem.,* 2014, **86**, 10131-10137, DOI: 10.1021/ac501932f.
17. J. Goldstein, D. Newbury, D. Joy, C. Lyman, P. Echlin, E. Lifshin, L. Sawyer and J. Michael, *Scanning Electron Microscopy and X-ray Microanalysis*, Springer, New York, USA, 3rd edn., 2003.
18. P. Comodi, S. Nazzareni, P. F. Zanazzi and S. Speziale, *Am. Mineral.,* 2008, **93**, 1530-1537, DOI: 10.2138/am.2008.2917.
19. H. Schmidt, I. Paschke, D. Freyer and W. Voigt, *Acta Crystallogr. B,* 2011, **67**, 467-475, DOI: 10.1107/s0108768111041759.
20. F. C. Hawthorne and R. B. Ferguson, *Canad. Mineral.,* 1975, **13**, 289-292, DOI: n/a.
21. B. N. Dutta, *Phys. Stat. Sol. B,* 1962, **2**, 984-987, DOI: 10.1002/pssb.19620020803.
